# UTX and UTY Demonstrate Histone Demethylase-Independent Function in Mouse Embryonic Development

**DOI:** 10.1371/journal.pgen.1002964

**Published:** 2012-09-27

**Authors:** Karl B. Shpargel, Toru Sengoku, Shigeyuki Yokoyama, Terry Magnuson

**Affiliations:** 1 Department of Genetics, Carolina Center for Genome Sciences, and Lineberger Comprehensive Cancer Center, The University of North Carolina at Chapel Hill, Chapel Hill, North Carolina, United States of America; 2 RIKEN Systems and Structural Biology Center, Tsurumi, Yokohama, Japan; 3 Laboratory of Structural Biology and Department of Biophysics and Biochemistry, Graduate School of Science, The University of Tokyo, Tokyo, Japan; Stanford University, United States of America

## Abstract

UTX (KDM6A) and UTY are homologous X and Y chromosome members of the Histone H3 Lysine 27 (H3K27) demethylase gene family. UTX can demethylate H3K27; however, *in vitro* assays suggest that human UTY has lost enzymatic activity due to sequence divergence. We produced mouse mutations in both *Utx* and *Uty*. Homozygous *Utx* mutant female embryos are mid-gestational lethal with defects in neural tube, yolk sac, and cardiac development. We demonstrate that mouse UTY is devoid of *in vivo* demethylase activity, so hemizygous X*^Utx−^* Y*^+^* mutant male embryos should phenocopy homozygous X*^Utx−^* X*^Utx−^* females. However, X*^Utx−^* Y*^+^* mutant male embryos develop to term; although runted, approximately 25% survive postnatally reaching adulthood. Hemizygous X*^+^* Y*^Uty−^* mutant males are viable. In contrast, compound hemizygous X*^Utx−^* Y*^Uty−^* males phenocopy homozygous X*^Utx−^* X*^Utx−^* females. Therefore, despite divergence of UTX and UTY in catalyzing H3K27 demethylation, they maintain functional redundancy during embryonic development. Our data suggest that UTX and UTY are able to regulate gene activity through demethylase independent mechanisms. We conclude that UTX H3K27 demethylation is non-essential for embryonic viability.

## Author Summary

Trimethylation at Lysine 27 of histone H3 (H3K27me3) establishes a repressive chromatin state in silencing an array of crucial developmental genes. Polycomb repressive complex 2 (PRC2) catalyzes this precise posttranslational modification and is required in several critical aspects of development including *Hox* gene repression, gastrulation, X-chromosome inactivation, mono-allelic gene expression and imprinting, stem cell maintenance, and oncogenesis. Removal of H3K27 trimethylation has been proposed to be a mechanistic switch to activate large sets of genes in differentiating cells. Mouse *Utx* is an X-linked H3K27 demethylase that is essential for embryonic development. We now demonstrate that *Uty*, the Y-chromosome homolog of *Utx*, has overlapping redundancy with *Utx* in embryonic development. Mouse UTY has a polymorphism in the JmjC demethylase domain that renders the protein incapable of H3K27 demethylation. Therefore, the overlapping function of UTX and UTY in embryonic development is due to H3K27 demethylase independent mechanism. Moreover, the presence of UTY allows UTX-deficient mouse embryos to survive until birth. Thus, UTX H3K27 demethylation is not essential for embryonic viability. These intriguing results raise new questions on how H3K27me3 repression is removed in the early embryo.

## Introduction

Post-translational modifications of histones establish and maintain active or repressive chromatin states throughout cell lineages. Thus, the enzymes that catalyze these modifications often have crucial roles in establishing genomic transcriptional states in developmental decision-making. Histone methylation can stimulate gene activation or repression depending on which residues are targeted. Methylation of histone H3 on Lysine 4 (H3K4me) is an active chromatin modification, while methylation on histone H3 Lysine 27 (H3K27me) is associated with repression of gene activity [1].

The polycomb repressive complex 2 (PRC2) methylates H3K27 [2], [3], [4], [5]. Within this complex, enhancer of zeste homolog 2 (EZH2) catalyzes di and tri-methylation of H3K27. Embryonic ectoderm development (EED) and suppressor of zeste homolog 12 (SUZ12) are additional PRC2 core components indispensible for PRC2 activity [6], [7], [8]. EZH1 is a secondary, less efficient H3K27 methyl-transferase that shares some overlapping redundancy with EZH2 in ES cells and epidermal stem cells [9], [10], [11], [12]. The PRC1 complex is recruited through H3K27 trimethylation for additional histone modification and chromatin compaction [13]. In embryonic stem (ES) cells, PRC2 targets and represses genes essential for developmental events [14], [15], [16], [17]. The promoters of these PRC2 targets typically contain “bivalent” chromatin marks with both active H3K4 and repressive H3K27 methylation [18], [19], [20]. Loss of PRC2 activity de-represses these genes but does not alter ES cell pluripotency [14]. However, mouse mutations in any of the three PRC2 core components are early embryonic lethal with gastrulation defects [7], [21],[22].

H3K27 trimethylation is reversible as a family of histone demethylases catalyzes the removal of this epigenetic mark [23], [24], [25], [26]. JMJD3 (KDM6B) is an autosomal H3K27 demethylase upregulated during specific differentiation events [25], [27]. UTX (KDM6A) is a broadly expressed X-linked H3K27 demethylase that escapes X-inactivation [23], [24], [26], [28]. UTY is the Y chromosome homolog of UTX. Both UTX and JMJD3 demethylate H3K27 di and tri-methyl residues; however, UTY lacks this activity *in vitro* [26], [29]. Based on cell culture models, UTX and JMJD3 mediated H3K27 demethylation is vital in a wide array of functions including cell cycle regulation, M2 macrophage differentiation, neuronal stem cell specification, skin differentiation, and muscle differentiation [27], [30], [31], [32], [33], [34], [35]. In contrast, the biological function of UTY remains unknown.

*Utx* and *Uty* are genetically amenable to delineate H3K27me3 demethylation dependent versus demethylation independent function in mouse development. Comparative amino acid sequence analysis of UTX and UTY reveals 88% sequence similarity in humans (83% identity) and 82% sequence similarity in mouse. Across the annotated JmjC histone demethylase domain, the similarity is at 98% and 97% for human and mouse respectively. In the TPR (tetratricopeptide repeat) domain, the similarity is at 94%. So while UTY is reported to have lost H3K27 demethylase activity, it is remarkably well conserved with respect to UTX. Recent discoveries have revealed that JMJD3 functions in macrophage lipopolysaccharide response and lymphocyte Th1 response through H3K27 demethylase independent gene regulation [36], [37], suggesting that function of this family of proteins is not limited to histone demethylation. It has been hypothesized that X and Y chromosome homologs will escape X-inactivation in instances where the Y homolog has not lost functional activity and male to female dosage remains balanced [38]. Therefore, it is possible that UTX and UTY have functional overlap in H3K27 demethylase independent gene regulatory processes.

A recent publication by Lee et al. characterized heart defects in *Utx* homozygous embryos [39]. Cell culture experiments suggested that the phenotype resulted from H3K27 demethylase activity. *Utx* hemizygotes were reported to have a wide range of abnormalities, but it was not clear if any phenotypes overlap with the *Utx* homozygotes as no comparative data were illustrated. Given that *Uty* remained intact in these studies, it was not possible to conclude definitively whether *Utx* demethylase activity was essential for early embryonic development. Furthermore, it is not known whether mouse UTY is capable of H3K27 demethylation. The classification of UTY as having no demethylase activity is based on *in vitro* assays only. The possibility of *in vivo* demethylase activity due to other co-factors remains a possibility. Also, mouse UTY has considerable sequence divergence from human UTY. The two proteins are 75% identical overall, and 95% identical in the JmjC demethylase domain. Thus, it is possible that mouse UTY has retained demethylase activity.

In our study, we have generated mouse mutations in both *Utx* and *Uty*. Hemizygous *Utx* mutant male mice (X*^Utx−^* Y*^+^*) were runted at birth with only a small number surviving to adulthood. In contrast, *Utx* homozygous females (X*^Utx−^* X*^Utx−^*) had severe phenotypes mid-gestation, with developmental delay, neural tube closure, yolk sac, and heart defects. Unlike homozygotes, *Utx* hemizygotes lack mid-gestational cardiovascular defects and are recovered in Mendelian frequencies at E18.5. Furthermore, compound hemizygous male embryos (X*^Utx−^* Y*^Uty−^*) carrying mutations of both *Utx* and *Uty* phenocopy the *Utx* homozygotes. Thus, the disparity in hemizygous and homozygous *Utx* phenotypes is due to compensation by *Uty* in the hemizygous male embryos. We have utilized an *in vivo* H3K27 demethylation assay to demonstrate that mouse UTY is not capable of H3K27 demethylation. Additionally, cell culture data indicate UTX and UTY may function in gene activation as both proteins associate with the H3K4 methyl-transferase complex, the BRG1 chromatin remodeler, as well as heart transcription factors. Our results implicate a crucial H3K27 demethylase independent function for UTX and UTY in mouse embryonic development. This is the first ascribed function for UTY, and the first example of developmental redundancy for X and Y chromosome homologous genes. Notably, our data suggest the H3K27 demethylase activity of UTX is not essential for embryonic viability.

## Results

### Hemizygous *Utx* mutant male mice have reduced peri-natal viability

**Figure 1:**
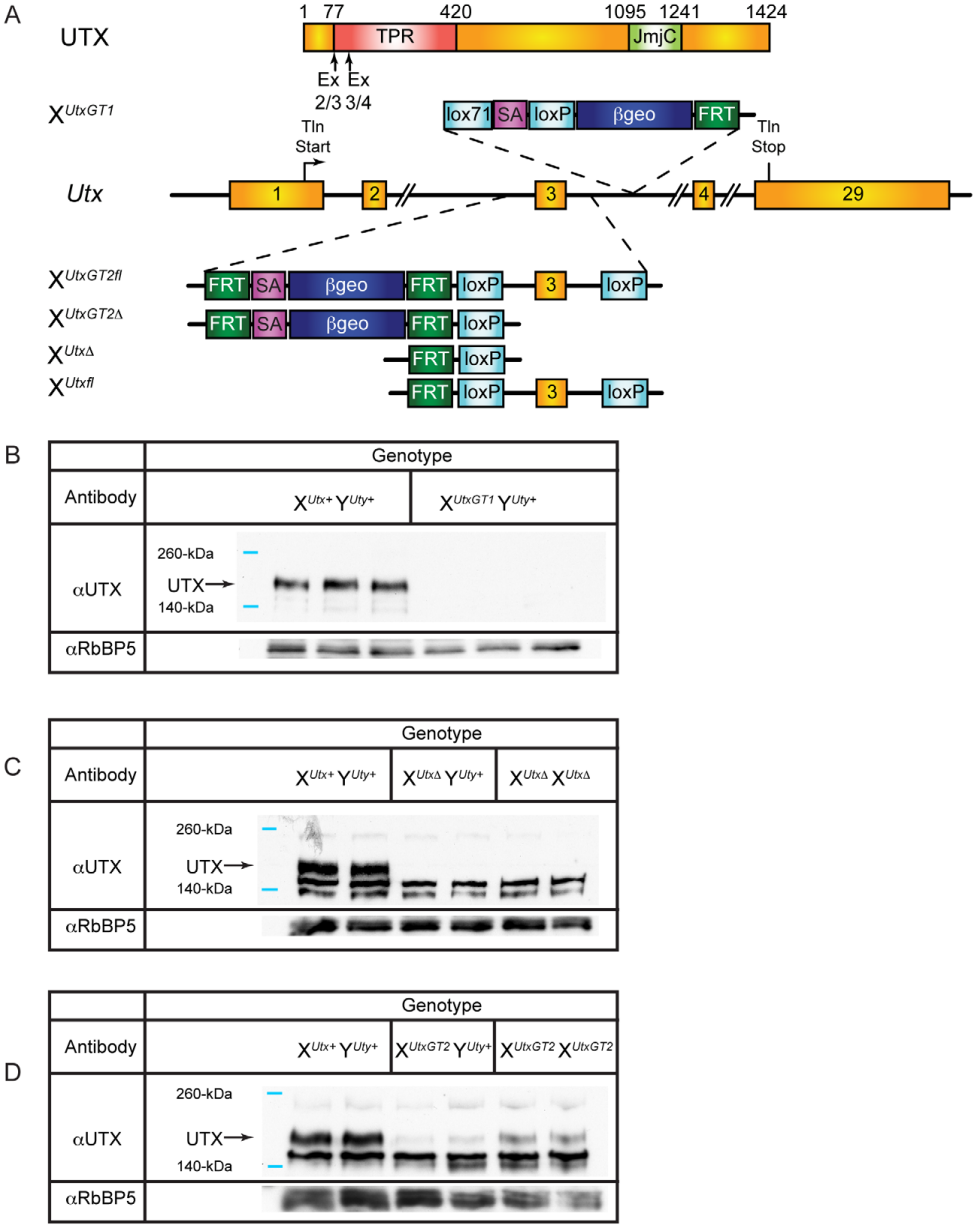
*Utx* mutant alleles. (A) Schematics of mouse mutations in *Utx*. Included are annotations and locations of where the protein would be mutated. Two *Utx* mutant alleles included a gene trap in intron 3 (X*^UtxGT1^*) and a gene trap/floxed exon 3 (X*^UtxGT2fl^*). A UTX protein annotation is illustrated at the top to indicate to positions of *Utx* alleles. A germline Cre recombinase deleted exon 3 in the X*^UtxGT2fl^* background to create X*^UtxGT2Δ^*. Additionally, the gene trap of X*^UtxGT2fl^* was excised with Flp recombinase to create a standard floxed exon 3 (X*^Utxfl^*) and Cre recombination created X*^UtxΔ^*. (B) Western blotting of E18.5 liver demonstrates a complete loss of UTX in X*^UtxGT1^* Y*^Uty+^* lysates. RbBP5 was used as a loading control. (C) Western blotting of E10.5 whole embryo demonstrates a complete loss of UTX in X*^UtxΔ^* Y*^Uty+^* and X*^UtxΔ^* X*^UtxΔ^* lysates. RbBP5 was used as a loading control. (D) Western blotting of E12.5 primary MEFs demonstrates a reduction of UTX in X*^UtxGT2fl^* Y*^Uty+^* and X*^UtxGT2fl^* X*^UtxGT2fl^* lysates. RbBP5 was used as a loading control.

We developed mutant mouse lines to assess the contribution of UTX H3K27 demethylase function in mouse development. Two alleles for *Utx* were obtained from public resources. The BayGenomics gene trap line *Kdm6a^Gt(RRA094)Byg^* is designated as X*^UtxGT1^* (Figure 1A). RT-PCR and PCR genotyping verified the identity of this allele in both ES cells and mutant mice (Figures S1 and S2A–S2C). Additionally, we obtained the EUCOMM *Kdm6a* knockout line (project 26585, *Kdm6a^tm1a(EUCOMM)Wtsi^*), designated as X*^UtxGT2fl^*, which inserts a gene trap in intron 2 along with a floxed 3^rd^ exon (Figure 1A). Southern blotting and PCR genotyping verified the identity of this allele (Figures S1 and S2D–S2F). Notably, quantitative RT-PCR comparison of tail RNA from X*^UtxGT1^* Y*^Uty+^* versus X*^UtxGT2fl^* Y*^Uty+^* mice demonstrated that *Utx* gene trap 1 is more effective than gene trap 2 (a 96% reduction compared to a 61% reduction in Figures S2C and S2F). Because X*^UtxGT2fl^* demonstrated incomplete trapping, the 3^rd^ exon was deleted with Cre recombinase to establish X*^UtxGT2Δ^* (containing both the gene trap and deleted 3^rd^ exon, Figure 1A). Deletion of the third *Utx* exon produces a frameshift and introduction of a translational stop codon when *Utx* is spliced from exon 2 to exon 4. X*^UtxGT1^* and X*^UtxΔ^* are null alleles as UTX protein was eliminated in western blotting of these embryonic lysates (Figure 1B, 1C). Consistent with RT-PCR data, X*^UtxGT2fl^* exhibits a reduction but not absence of UTX protein (Figure 1D).

**Figure 2:**
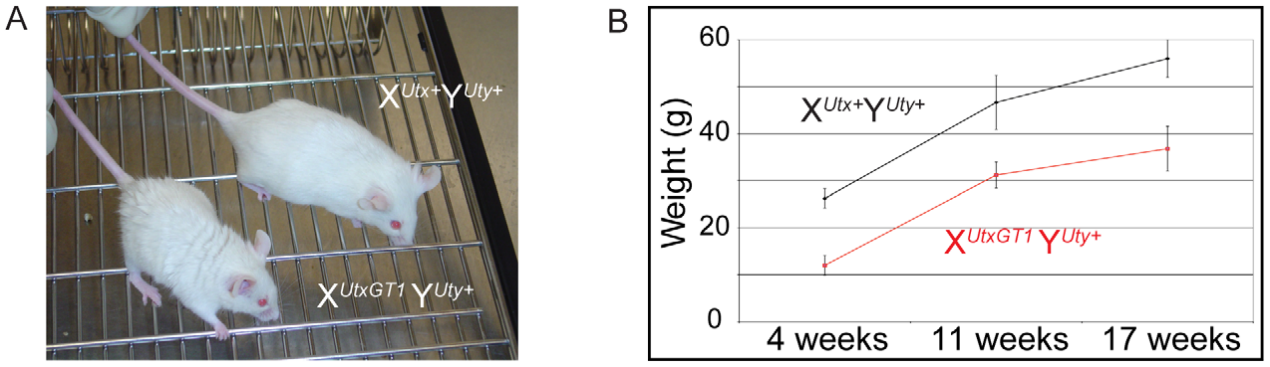
Hemizygous male *Utx* mutant mice are runted. (A) Hemizygous male *Utx* mutant mice are runted in size. Wild type male X*^Utx^* ^+^ Y^+^ mice are displayed next to hemizygous X*^UtxGT1^* Y^+^ mice. (B) The hemizygous mice exhibit a smaller size throughout adulthood.

Heterozygous *Utx* female mice were crossed to wild type male mice to produce hemizygous *Utx* mutant males. At weaning, the hemizygous X*^UtxGT1^* Y*^Uty+^*, X*^UtxGT2Δ^* Y*^Uty+^*, and X*^UtxGT2fl^* Y*^Uty+^* mice all exhibited reductions of 68%, 83%, and 55% respectively from the expected genotype frequencies based on these crosses, yet expected genotype frequencies were observed at embryonic day E18.5 (Table 1). At E18.5, most of the hemizygous *Utx* males appeared phenotypically normal; however a small percentage of the fetuses exhibited exencephaly. At birth, the hemizygous *Utx* males were small and exhibited a failure to thrive phenotype. Those males that survived through this phenocritical phase reached adulthood and were fertile. Hemizygous *Utx* mutant males were runted compared to wild type littermates and remained smaller than controls throughout their lifespan (Figure 2A, 2B). Backcross of the *Utx* allele onto a C57BL/6J or 129/SvJ background affected postnatal viability, but hemizygous *Utx* male embryos were still readily obtained at E18.5 (Table S1).

**Table 1:** Genotype frequencies of *Utx* and *Uty* mutant mice.

Genotype frequencies of *Utx* hemizygous mutant males						
Geno:	X*^UtxGT1^* Y*^Uty+^*		X*^UtxGT2Δ^* Y*^Uty+^*		X*^UtxGT2fl^* Y*^Uty+^*	
Stage:	Obs(Ex)	p-value	Obs(Ex)	p-value	Obs(Ex)	p-value
E10.5	42(47)	0.43	60(60)	0.38	-	-
E18.5	26(30)	0.30	31(22)	0.05	-	-
P25	27(85)	0.00	3(10)	0.10	14(31)	0.00

Genotype frequencies of *Utx* homozygous mutant females						
Geno:	X*^UtxGT1^* X*^UtxGT1^*		X*^UtxGT2Δ^* X*^UtxGT2Δ^*		X*^UtxGT2fl^* X*^UtxGT2fl^*	
Stage:	Obs(Ex)	p-value	Obs(Ex)	p-value	Obs(Ex)	p-value
E10.5	47(42)	0.62	58(60)	0.38	-	-
E12.5	0(11)	0.00	0( 6)	0.04	17(25)	0.04
E14.5	-	-	-	-	0( 8)	0.01
E18.5	0( 9)	0.01	0(20)	0.00	-	-
P25	0(21)	0.00	-	-	-	-

Genotype frequencies of *Uty* hemizygous mutant males and *Utx/Uty* compound hemizygous mutant males						
Geno:	X*^Utx+^* Y*^UtyGT^*		X*^UtxGT1^* Y*^UtyGT^*		X*^UtxGT2Δ^* Y*^UtyGT^*	
Stage:	Obs(Ex)	p-value	Obs(Ex)	p-value	Obs(Ex)	p-value
E10.5	-	-	3( 3)	0.97	33(36)	0.42
E18.5	-	-	0(29)	0.00	0(17)	0.00
P25	40(47)	0.18	-	-	-	-

Observed (Obs) and expected (Ex) frequencies of indicated genotypes (Geno) at embryonic (E) or postnatal (P) developmental stages with χ^2^ p-values (p-value) for the corresponding crosses to obtain each genotype.

### Homozygous *Utx* females are mid-gestational embryonic lethal

**Figure 3:**
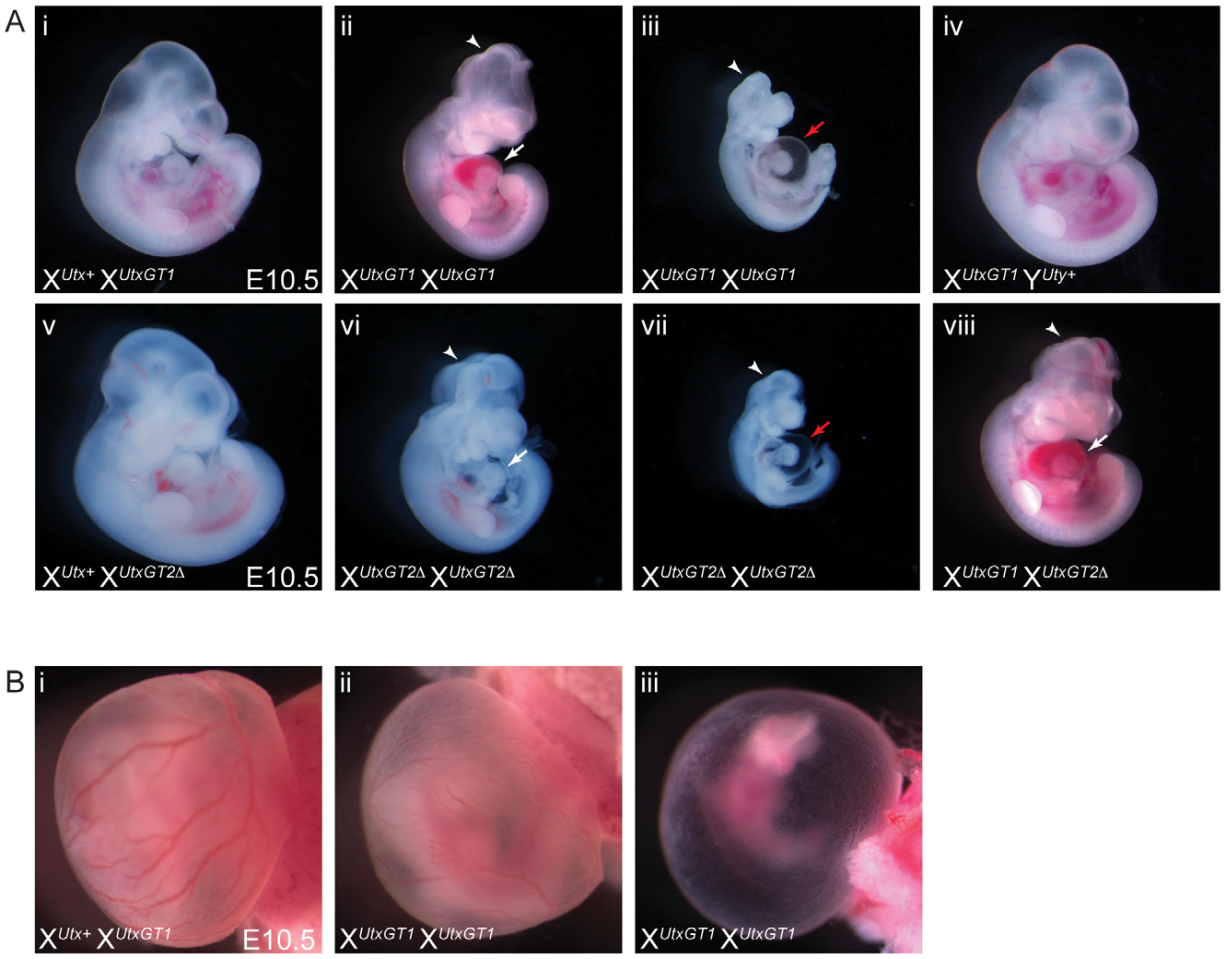
Homozygous female *Utx* mutant embryos have mid-gestational developmental delay. (A) Compared to controls (A-i and A-v), homozygous female E10.5 X*^UtxGT1^* X*^UtxGT1^* (A-ii) and X*^UtxGT2Δ^* X*^UtxGT2Δ^* (A-vi) embryos have some developmental delay including smaller size, underdeveloped hearts (white arrows), and open neural tube in the head (arrowheads). More severe embryos resemble the size and features of E9.5 embryos with cardiac abnormalities and peri-cardial edema (A-iii, vii, red arrows). Hemizygous male X*^UtxGT1^* Y^Uty+^ embryos appear phenotypically normal at this stage (A-iv). The X*^UtxGT1^* and X*^UtxGT2Δ^* alleles fail to complement as female X*^UtxGT1^* X*^UtxGT2Δ^* embryos have identical phenotypes to homozygotes (A-viii). (B) At E10.5, homozygous X*^UtxGT1^* X*^UtxGT1^* female embryos exhibit either normal yolk sac vasculature with a reduction in red blood cells (B-ii) or have a completely pale yolk sac with unremodeled vascular plexus (B-iii).

Human UTY lacks demethylase activity based on *in vitro* assays, so we hypothesized that X*^Utx−^* X*^Utx−^* homozygous females will phenocopy X*^Utx−^* Y*^Uty+^* hemizygous males in demethylase dependent function (UTX specific), but may demonstrate a more severe phenotype in demethylase independent roles. Homozygous X*^UtxGT1^* X*^UtxGT1^* and X*^UtxGT2Δ^* X*^UtxGT2Δ^* females were never observed at weaning or embryonic day E18.5 (Table 1), but were observed at expected genotype frequencies at E10.5. However, these embryos were dead and resorbed by E12.5 (Table 1). Notably, at E10.5 all homozygous X*^UtxGT1^* X*^UtxGT1^* and X*^UtxGT2Δ^* X*^UtxGT2Δ^* females were smaller in size and had open neural tubes in the midbrain region (Figure 3A-ii, iii, vi, vii). Variation in severity of the *Utx* homozygous phenotypes was observed in mutant embryos, ranging from medium sized with typical E10.5 features (Figure 3A-ii, vi) to much smaller embryos resembling the E9.5 timepoint (Figure 3A-iii, vii). The X*^UtxGT1^* and X*^UtxGT2Δ^* alleles failed to complement, as trans-heterozygous X*^UtxGT1^* X*^UtxGT2Δ^* female embryos resembled individual homozygous alleles (Figure 3A-viii). Hemizygous X*^UtxGT1^* Y*^Uty+^* male embryos appeared phenotypically normal at E10.5 (Figure 3A-iv). Homozygous X*^UtxGT2fl^* X*^UtxGT2fl^* females exhibited a slight reduction in phenotypic severity; about half of the mutant embryos had open neural tubes and some survival to E12.5 (Table 1). To distinguish between embryonic and extraembryonic contribution of UTX towards the homozygous phenotype, we crossed the *Sox2Cre* transgene into the *Utx^fl^* background. In this cross, paternally inherited *Sox2Cre* expression will drive *Utx* deletion specifically in embryonic tissue [40]. No X*^Utxfl^* X*^Utxfl^*, *Sox2Cre* female embryos were recovered at E18.5, whereas X*^Utxfl^* Y*^Uty+^*, *Sox2Cre* male embryos were recovered at expected frequencies (Table S2). At E10.5, X*^Utxfl^* X*^Utxfl^*, *Sox2Cre* embryos produced phenotypes largely identical to *Utx* homozygotes. In summary, *Utx* homozygous females demonstrate a significantly more severe embryonic phenotype in comparison to *Utx* hemizygous males.

Mid-gestational lethality is typically associated with defective cardiovascular development. Accordingly, we observed both heart and yolk sac vasculature/hematopoietic phenotypes in *Utx* homozygotes. *Utx* homozygous mutant hearts were small and underdeveloped, and more severe embryos exhibited peri-cardial edema (Figure 3A-ii, iii, vi, vii). The yolk sac vasculature of *Utx* homozygotes was pale with a reduction in the amount of vascular blood (Figure 3B-ii). In more severe examples, homozygous yolk sacs were completely pale with an unremodeled vascular plexus (Figure 3B-iii). Thus, abnormal cardiovascular function may be a source of lethality and developmental delay in *Utx* homozygous mutant embryos.

### UTX and UTY have redundant function in embryonic development

The most likely explanation for the disparity between *Utx* hemizygotes and homozygotes is that UTY can compensate for the loss of UTX in embryonic development. We tested *Utx* and *Uty* expression in embryonic development to assess any overlap in expression patterns. *Utx* expression was initially gauged utilizing the B-galactosidase reporter in X*^Utx+^* X*^UtxGT1^* and X*^UtxGT1^* X*^UtxGT1^* whole mount E10.5 embryos. *Utx* was expressed at lower levels throughout the E10.5 embryo with a particular enrichment in the neural tube and otic placode (Figure S3A-ii, iii, iv). *In situ* hybridization for both *Utx* and *Uty* demonstrated similar expression patterns characterized by widespread low-level expression with particular enrichment in the neural tube (Figure S3B-ii, iii, v, vi). Our analysis of publicly available RNA-seq data sets [41], [42] revealed similar low-levels of expression for *Utx* and *Uty*.

**Figure 4:**
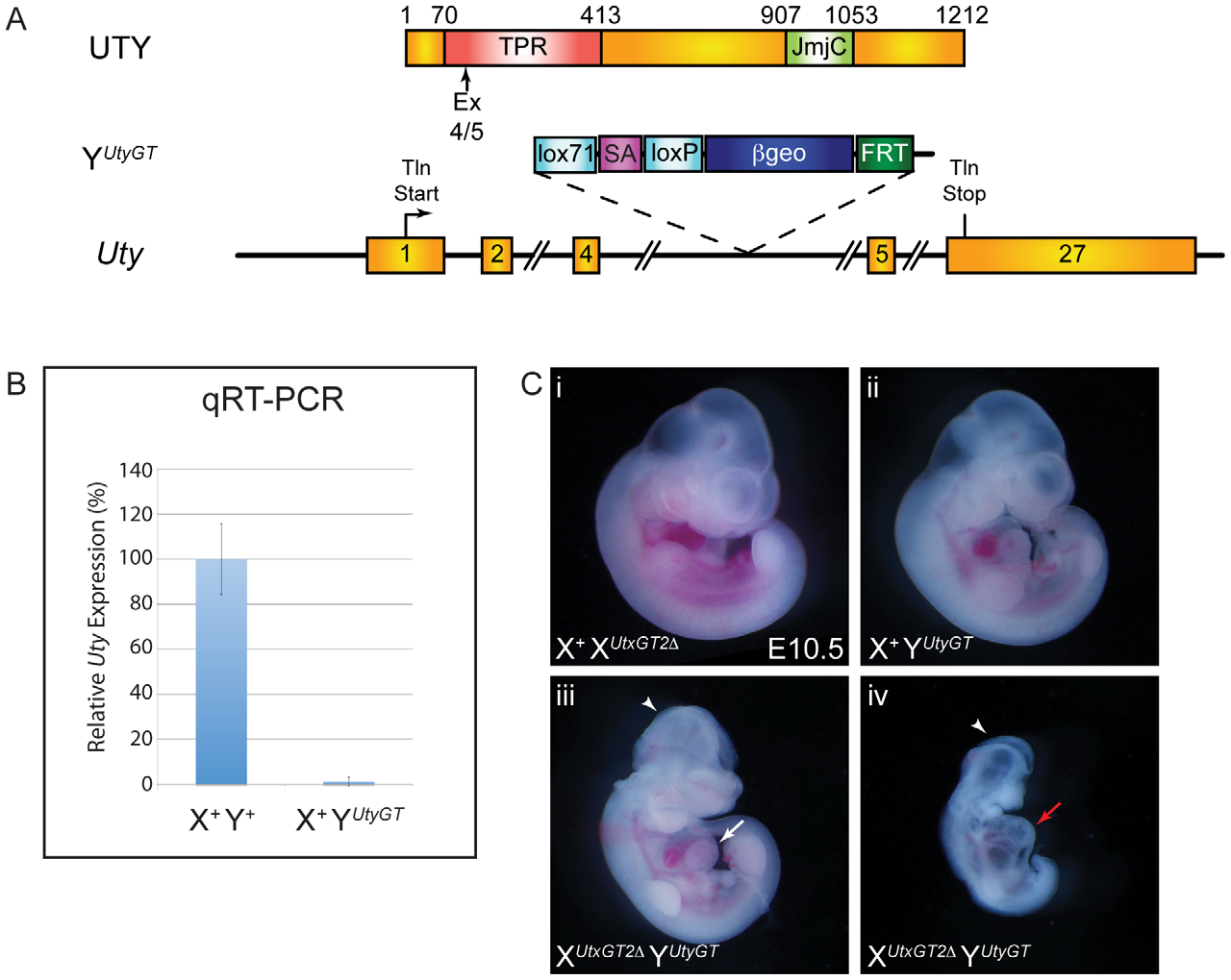
UTX and UTY have essential, redundant functions in embryonic development. (A) Schematic of mouse mutation in *Uty*. The *Uty* gene trap Y*^UtyGT^* is located in intron 4. Protein annotation is illustrated at the top to denote the location of the gene trap within the *Uty* coding sequence. (B) Quantitative RT-PCR downstream of the gene trap (exon 15) from tail RNA of X^+^ Y^UtyGT^ mice demonstrates essentially no mutant RNA. (C) X*^UtxGT2Δ^* Y*^UtyGT^* males (C-iii, iv) have identical phenotypes to X*^UtxGT2Δ^* X*^UtxGT2Δ^* females (Figure 3A-vi, vii). Arrowheads denote open neural tube in the head, while white and red arrows denote moderate and more severe cardiac phenotypes.

To determine whether UTY can compensate for the loss of UTX, we obtained the Welcome Trust Sanger Institute gene trap line *Uty^Gt(XS0378)Wtsi^*, designated as Y*^UtyGT^* (Figure 4A). This line, inserted in intron 4, traps the *Uty* transcript in a similar position of the coding sequence as the *Utx* alleles (compare to Figure 1A). This gene trap line was verified by RT-PCR in ES cells and subsequent mice (Figures S1 and S2G), and it achieved a 99% reduction in *Uty* expression from X*^Utx+^* Y*^UtyGT^* mouse tail RNA (Figure 4B). Hemizygous *Uty* mutant males, X*^Utx+^* Y*^UtyGT^*, were viable and fertile (Table 1). However, no compound hemizygous X*^UtxGT1^* Y*^UtyGT^* and X*^UtxGT2Δ^* Y*^UtyGT^* embryos were recovered at E18.5 (Table 1). At E10.5, expected genotype frequencies of X*^UtxGT1^* Y*^UtyGT^* and X*^UtxGT2Δ^* Y*^UtyGT^* males were observed, but these embryos phenocopied the developmental delay, neural tube closure, cardiac, and yolk sac defects observed in *Utx* homozygous embryos (Figure 4C-iii, iv).

### UTX and UTY redundancy is essential for progression of cardiac development

**Figure 5:**
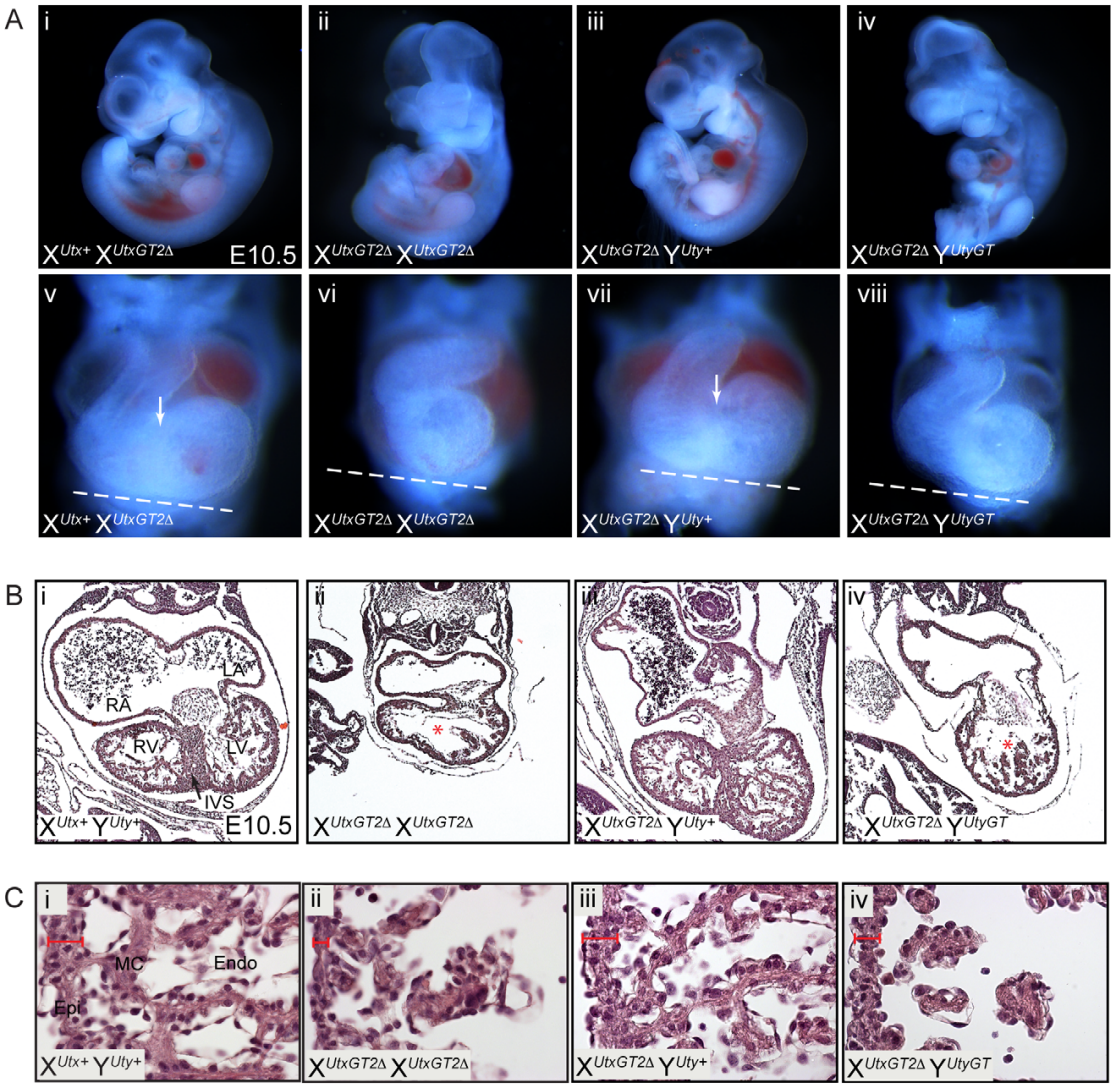
UTX and UTY redundancy is essential for progression of cardiac development. (A) Similar sized *Utx* heterozygous (i), *Utx* homozygous (ii), *Utx* hemizygous (iii), or *Utx/Uty* compound hemizygous (iv) embryos were analyzed in more detail for cardiac developmental abnormalities. Frontal views of the respective hearts of these embryos revealed that *Utx* homozygotes and *Utx/Uty* compound hemizygotes (A-vi, viii) have smaller hearts that have not completed looping relative to *Utx* heterozygotes (A-v) or hemizygotes (A-vii). A white dashed line was drawn at an identical angle in all panels to illustrate the failure of hearts to loop around in alignment with this appropriate plane. Only control and *Utx* hemizygous embryos (A-v, vii) have initiated the formation of the interventricular groove (white arrows), indicative of early interventricular septum development. (B) Transverse sections of E10.5 X*^UtxGT2Δ^* X*^UtxGT2Δ^* (B-ii) and X*^UtxGT2Δ^* Y*^UtyGT^* (B-iv) embryos reveal smaller heart size with defects in ventricular myocardial trabeculation and organization. The control and *Utx* hemizygous hearts initiated the formation of the interventricular septum (B-i, iii, IVS, black arrow), while other mutant combinations (B-ii, iv) have not (red asterisk). RA and LA = Right and Left Atrium, and RV and LV = Right and Left Ventricle. (C) More magnified images further illustrate the narrowing of the ventricular wall (red scale) and the lack of myocardial cells and structure in *Utx* homozygotes and *Utx/Uty* compound hemizygotes (Epi = epicardium, MC = myocardium, Endo = Endocardium).

We performed a more detailed phenotypic assessment of *Utx* and *Uty* mutant hearts to scrutinize the extent of phenotypic overlap between X*^Utx−^* Y*^Uty+^*, X*^Utx−^* X*^Utx−^*, and X*^Utx−^* Y*^Uty−^* embryos. Analysis of cardiac development in similar sized E10.5 embryos (Figure 5A-i, ii, iii, iv) revealed that *Utx* homozygotes and *Utx/Uty* compound hemizygotes failed to complete heart looping (Figure 5A-vi, viii), whereas *Utx* heterozygotes and hemizygotes were phenotypically normal (Figure 5A-v, vii). Additionally, homozygotes and compound hemizygotes had smaller hearts with a lack of constriction between the left and right ventricles. Sectioning of E10.5 hearts confirmed that *Utx* homozygotes and *Utx*/*Uty* compound hemizygotes have small hearts with a reduction in ventricular myocardial trabeculation and little or no initiation of interventricular septum formation (Figure 5B-ii, iv). The outer ventricular wall of these embryos is much thinner, and the overall number of cardiomyocytes and myocardial structure is severely deficient (Figure 5C-ii, iv). In summary, while mid-gestational hearts appear normal in X*^Utx−^* Y*^Uty+^* hemizygous males, X*^Utx−^* X*^Utx−^*homozygous females and X*^Utx−^* Y*^Uty−^* compound hemizygous males display identical deficiencies in cardiac development. Therefore, UTY compensates for the loss of UTX in hemizygous *Utx* mutant males, rescuing mid-gestational cardiac phenotypes.

### Mouse and human UTY are incapable of H3K27 demethylation *in vivo*

**Figure 6:**
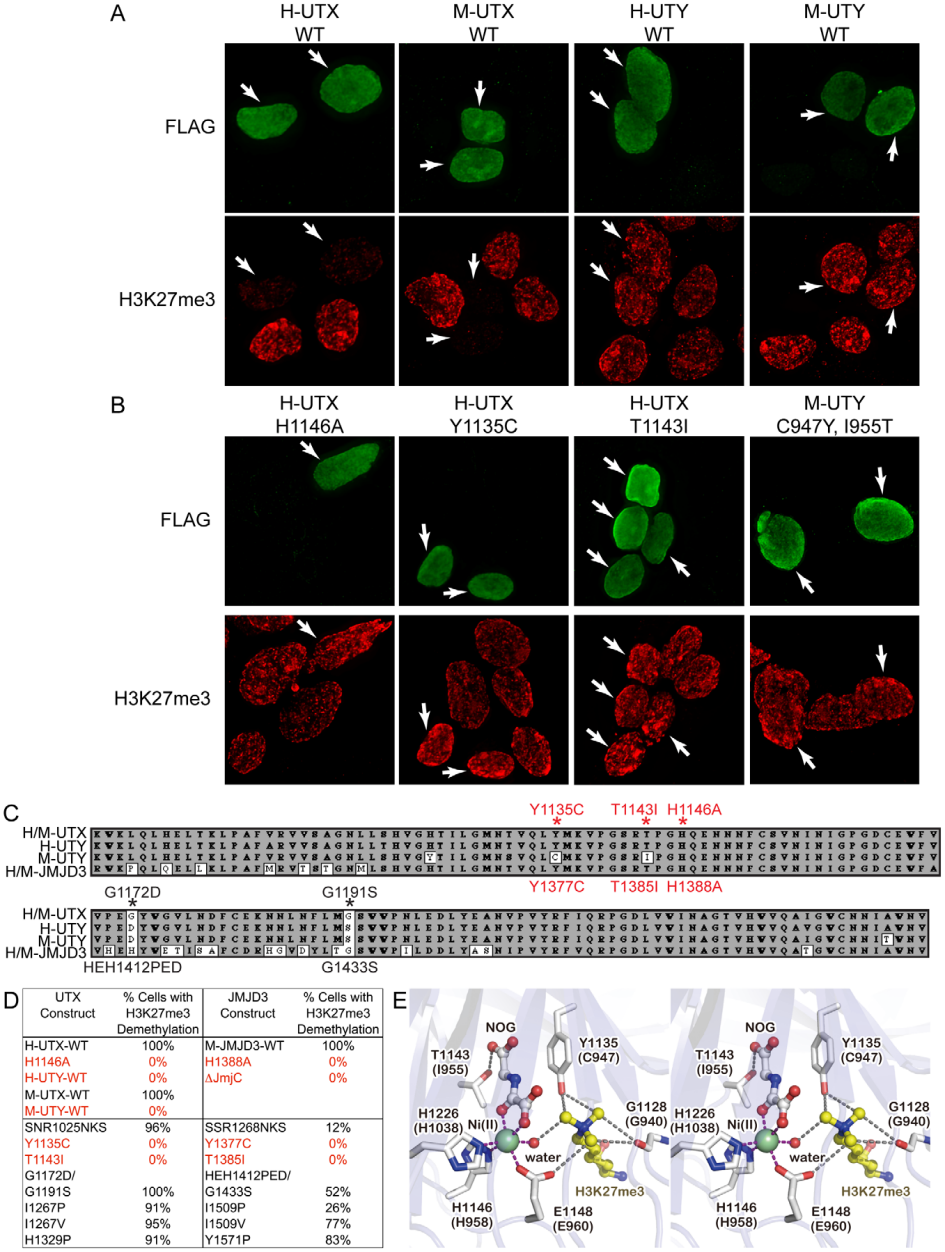
Human and mouse UTY have no H3K27 demethylase activity. (A) HEK293T cells were transfected with Flag-tagged C-terminal human (H) and mouse (M) UTX and UTY constructs. The C-terminal fragments span AA 880–1401 in human UTX (Figure S6) and include the corresponding regions in mouse UTX. Transfected cells (white arrows) over-expressing H-UTX and M-UTX (Flag immunofluorescence, green pseudo-color) exhibited global loss of H3K27me3 immunofluorescence (red pseudo-color). Cells transfected with H-UTY and M-UTY C-terminal constructs did not demethylate H3K27me3. (B) H3K27me3 demethylase assay of UTX and UTY mutant constructs. H-UTX H1146A contains a point mutation in a residue that was previously reported as defective in H3K27 demethylation. Cells expressing H-UTX H1146A had no loss of H3K27me3. Mouse UTY has a Y to C amino acid change that corresponds to position 1135 in human UTX. This UTX residue is predicted to regulate H3K27me3 binding and demethylation. Expression of H-UTX Y1135C failed to demethylate H3K27me3. Mouse UTY also has a T to I amino acid change that corresponds to position 1143 in human UTX that is predicted to regulate binding of ketoglutarate in the demethylase reaction. Expression of H-UTX T1143I failed to demethylate H3K27me3. Correction of these two altered residues in mouse Uty (M-UTY-C947Y, I955T) failed to recover H3K27me3 demethylase activity. (C) Alignment of the JmjC domains of human/mouse UTX human UTY, mouse UTY, and human/mouse JMJD3. UTY non-conservative substitutions are indicated by white boxes and residues of interest are labeled with red asterisks. The UTX mutations that were analyzed are listed above the alignment, while JMJD3 mutations are listed below the alignment. (D) HEK293T cells were transfected with C-terminal UTX and UTY constructs or full-length mouse JMJD3 constructs carrying various AA substitutions. Medium-high expressing cells (N≥100 cells scored for each experiment) were scored for any visible reduction in H3K27me3 levels relative to nearby untransfected cells. 100% of WT H-UTX, M-UTX and M-JMJD3 expressing cells had observable H3K27me3 demethylation. The negative controls of H-UTX H1146A, M-JMJD3 H1388A, and M-JMJD3 with deletion of the JmjC domain had no visible H3K27me3 demethylation (0% of cells). Wild type H-UTY and M-UTY had 0% of cells with detectable demethylation. Of the point mutations in UTY predicted to affect H3K27me3, only mutation of H-UTX Y1135C and T1143I with corresponding M-JMJD3 Y1377C and T1385I had no cells with any detectable H3K27 demethylation (0%). (E) Stereo view of the active site of human UTX (PDB ID: 3AVR). The corresponding residues in mouse UTY are also indicated in parentheses. The figure was prepared with the program Pymol (Schrodinger LLC).

UTX and UTY have redundant function in embryonic development, but it is not known whether mouse UTY is capable of H3K27 demethylation. Two independent publications demonstrated that human UTY has no catalytic activity in H3K27 demethylation *in vitro* [26], [29]. It is possible that human UTY (and not mouse UTY) has accumulated a specific polymorphism rendering it demethylase deficient. Additionally, *in vitro* assays remove UTY from its natural cellular context and may lack co-factors required to promote H3K27 demethylation. Therefore, we utilized an intracellular, *in vivo* demethylation assay, whereby HEK293T cells transiently over-expressing the UTX carboxy-terminus (encoding the JmjC and surrounding domains essential for proper structure and function) exhibit a reduction in H3K27me3 immunofluorescence levels [43]. In our assay, wild type and mutant constructs were expressed at similar levels (Figure S4A), and individual cells expressing similar, medium-high expression levels of each construct were selected for analysis (Figure 6). Expression of Flag-tagged human and mouse UTX demethylated H3K27me3 and H3K27me2, while a mutation known to disrupt activity (H1146A) was unable to demethylate H3K27 (Figure 6A and 6B, Figure S5A). Human UTX expression had no effect on other histone modifications we tested, such as H3K4me2 (Figure S5B). In contrast, neither human nor mouse UTY were capable of demethylating H3K27me3 and H3K27me2 (Figure 6A and Figure S5A). Cells expressing medium-to-high levels of UTY (N>100) never exhibited a reduction in H3K27me3 levels relative to nearby untransfected controls.

Our previous structural analysis of human UTX [43], combined with sequence alignments (Figure 6C and Figure S6), suggested several amino acid substitutions in human and mouse UTY sequences might make them catalytically inactive. We introduced these mutations into the human UTX C-terminal fragment (Y1135C, T1141I, SNR1025NKS, G1172D/G1191S, I1267P, I1267V, and H1329P), and examined their effects on the *in vivo* demethylation activity. Of all the mutations tested, only the Y1135C and T1143I mutations completely abolished the ability of UTX to demethylate H3K27 (Figure 6B, 6D). Complete loss of activity was similarly caused by mutations of the corresponding residues in JMJD3 (Y1377C and T1385I, Figure 6D). All qualitative data was also confirmed by immunofluorescence quantification (Figure S4B). Y1135 is conserved throughout all H3K27 demethylases (Figure S7), and in the crystal structure [43], it interacts with two of the three methyl groups of the H3K27me3 side chain, as well as N-oxalylglycine (NOG; an analog of the cofactor alpha-ketoglutarate) (Figure 6E). The smaller C947 side chain of mouse UTY would not effectively maintain either interaction. T1143 is conserved throughout H3K27, H3K9, and H3K36 demethylases (Figure S8), and also interacts with NOG (Figure 6E). Its replacement with bulky isoleucine not only removes the hydroxyl group for interaction with alpha-ketoglutarate, but also may sterically hinder its binding. These observations are consistent with the fact that no H3K27 demethylation activity has been detected for mouse UTY, and we therefore conclude that the catalytic domain of mouse UTY has crucial amino acid replacements that render the protein incapable of H3K27 demethylation. On the other hand, we failed to identify why human UTY is catalytically inactive. Notably, restoring the 2 crucial mouse UTY polymorphisms (M-UTY C947Y, I955T) failed to recover H3K27 demethylase activity (Figure 6B). These data suggest that unidentified structural elements in the UTY C-terminal region are also responsible for the lack of H3K27 demethylase activity.

### UTX and UTY associate in common protein complexes and are capable of H3K27 demethylase independent gene regulation

**Figure 7:**
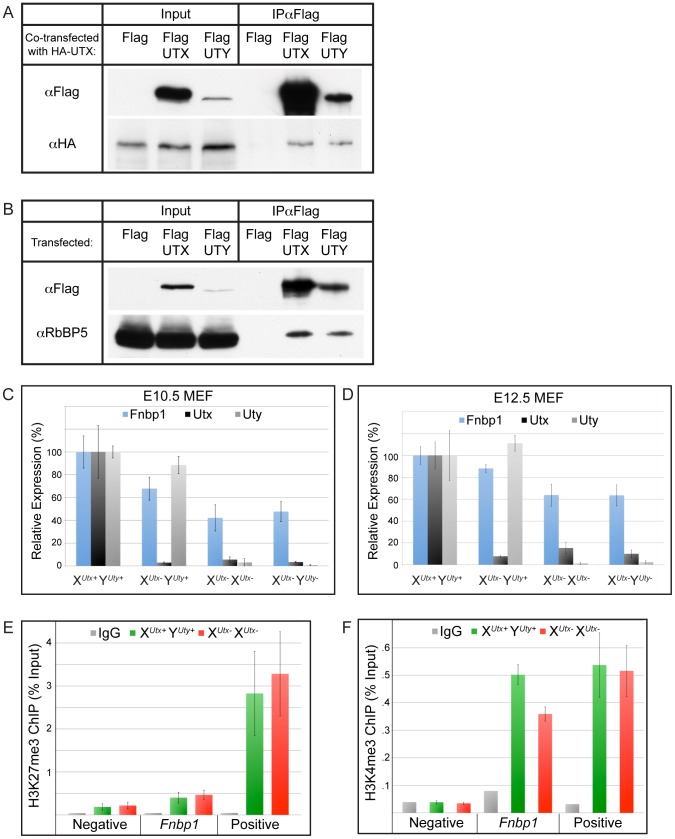
UTX and UTY associate in common protein complexes and are capable of H3K27 demethylase independent gene regulation. (A) Co-transfection of HA-UTX with Flag-UTX or Flag-UTY demonstrates that HA-UTX can immunoprecipitate with both Flag-UTX and Flag-UTY. (B) Immunoprecipitation of Flag-UTX and Flag-UTY reveal interaction with RBBP5, a component of the H3K4 methyl-transferase complex. Flag vector transfection was used as a negative control for immunoprecipitation. (C) *Fnbp1*, a gene targeted directly by UTX, has intermediate downregulation in X*^Utx−^* Y*^Uty+^* MEFs (68% of WT, t-test p-value = 0.002), but was further compromised in X*^Utx−^* X*^Utx−^* (42% of WT, t-test p-value relative to X*^Utx−^* Y*^Uty+^* = 0.001) and X*^Utx−^* Y*^Uty−^* (48% of WT, t-test p-value relative to X*^Utx−^* Y*^Uty+^* = 0.02, N>4 independent MEF lines per genotype) MEFs. MEFs were generated from the X*^UtxGT2Δ^* and Y*^UtyGT^* alleles. (D) *Fnbp1* is similarly mis-expressed in X*^UtxGT2fl^* allelic combinations of E12.5 MEFs. X*^Utx−^* X*^Utx−^* and X*^Utx−^* Y*^Uty−^* MEFs significantly differ from X*^Utx−^* Y*^Uty+^* MEFs (t-test p-value = 0.05 and 0.02 respectively, N>4 independent MEF lines per genotype). (E) H3K27me3 ChIP was performed on E12.5 X*^Utx+^* Y*^Uty+^* control (green) and X*^Utx−^* X*^Utx−^* (red) MEFs. An IgG antibody control is indicated in grey. Quantitative PCR for the ChIP was performed over a negative control region (an intergenic region) as well as a positive control (*HoxB1*). *Fnbp1* failed to accumulate H3K27me3 in X*^Utx−^* X*^Utx−^* MEFs (t-test p-value = 0.5, N = 4 independent MEF lines per genotype). (F) H3K4me3 ChIP was performed on E12.5 X*^Utx+^* Y*^Uty+^* control (green) and X*^Utx−^* X*^Utx−^* (red) MEFs. An IgG antibody control is indicated in grey. Quantitative PCR for the ChIP was performed over a negative control region (intergenic region) as well as a positive control (*Npm1*). The WT *Fnbp1* promoter exhibited significant H3K4me3 accumulation, which was reduced in X*^Utx−^* X*^Utx−^* MEFs (t-test p-value = 0.005, N = 3 independent MEF lines per genotype).

Although human and mouse UTY have lost the ability to demethylate H3K27, they retain considerable sequence similarity with UTX, suggesting a conserved function. To gain more insight into the overlap in UTX and UTY activities, we performed a biochemical analysis of tagged constructs to determine if UTX and UTY can associate in common protein complexes. Co-transfection of Flag tagged UTX or UTY with HA-UTX followed by immunoprecipitation demonstrates that UTX can form a multimeric complex with itself and UTY (Figure 7A). UTX associates with a H3K4 methyl-transferase complex containing MLL3, MLL4, PTIP, ASH2L, RBBP5, PA-1, and WDR5 [23], [44]. To examine incorporation into this complex, we performed immunoprecipitations with Flag tagged UTX and UTY constructs. Both UTX and UTY were capable of associating with RBBP5 (Figure 7B). Thus, UTX and UTY are incorporated into common protein complexes.

To identify common gene targets of UTX and UTY mediated regulation we generated E10.5 mouse embryonic fibroblast (MEF) cell lines containing mutations in *Utx* and *Uty* (alleles X*^UtxGT2Δ^* and Y*^UtyGT^*). The gene traps in these MEFs efficiently trapped *Utx* and *Uty* transcripts (Figure 7C). These MEFs did not demonstrate differences in levels of global H3K27me3 (Figure S9A). Genome-wide UTX promoter occupancy has been mapped in fibroblasts [30]. Therefore, we screened our mutant MEFs for misregulated genes affected by the loss of both *Utx* and *Uty* that had been documented as direct UTX targets. The *FNBP1* promoter is bound by UTX [30]. We verified UTX and UTY binding to the *Fnbp1* promoter by ChIP (Figure S9B and S9C). *Fnbp1* expression was reduced to 68% of WT levels in X*^Utx−^* Y*^Uty+^* MEFs, but was further compromised to 42% in X*^Utx−^* X*^Utx−^* lines and 48% in X*^Utx−^* Y*^Uty−^* MEFs in which all *Utx* and *Uty* activity was lost (Figure 7C). Analysis of E12.5 MEFs of a secondary allele (X*^UtxGT2fl^*) also demonstrated diminished *Fnbp1* expression in both X*^Utx−^* X*^Utx−^* and X*^Utx−^* Y*^Uty−^* MEFs (Figure 7D). Therefore, *Fnbp1* expression is positively regulated by both UTX and UTY.

To examine the role of UTX and UTY in *Fnbp1* regulation, we performed H3K27me3 ChIP on E12.5 X*^Utx+^* Y*^Uty+^* or X*^Utx−^* X*^Utx−^* MEFs (Figure 7E). Quantitative PCR for an intergenic region served as a negative control, while *HoxB1* served as a positive control for H3K27me3. Quantitative PCR demonstrated that the *Fnbp1* promoter has relatively low levels of H3K27me3 with no additional accumulation in X*^Utx−^* X*^Utx−^* MEFs (Figure 7E). Alternatively, H3K4me3 significantly accumulated at the *Fnbp1* promoter (Figure 7F). Notably, a loss of *Fnbp1* H3K4me3 was observed in X*^Utx−^* X*^Utx−^* MEFs (Figure 7F). Therefore, UTX and UTY appear to function in *Fnbp1* activation by regulating promoter H3K4 methylation rather than H3K27 demethylation.

### UTX and UTY can both associate with heart transcription factors to regulate downstream target genes

**Figure 8:**
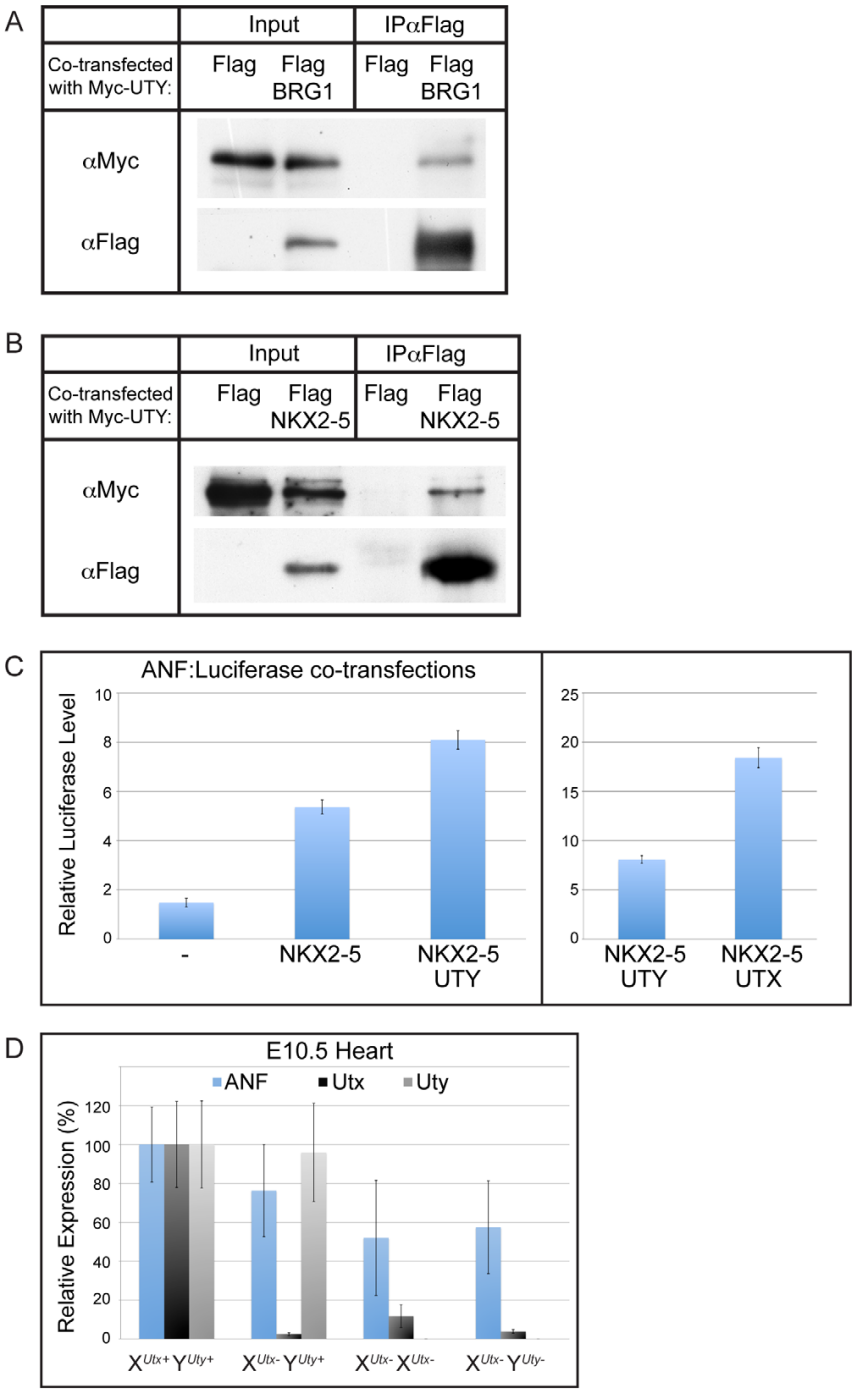
UTY associates with BRG1 and heart transcription factors, and regulates downstream ANF gene expression. (A) Myc-UTY was co-transfected with a Flag vector control or Flag BRG1 and immunoprecipitated with Flag-Agarose beads. Myc-UTY specifically immunoprecipitates with Flag-BRG1. (B) Myc-UTY was co-transfected with the Flag vector control or Flag-NKX2–5. Myc-UTY was co-immunoprecipitated by Flag-NKX2–5. (C) ANF:Luciferase reporter assay. HEK293T were transfected with the reporter ANF:Luciferase construct alone (-), with NKX2–5, with NKX2–5 and UTY, or with NKX2–5 and UTX. Reporter activity was significantly enhanced with the addition of UTY (t-test p-value = 0.01). Right panel illustrates the comparison of UTY versus UTX enhancement of NKX2–5 driven ANF expression. N = 3 independent transfections per group. (D) *Anf* expression was analyzed from E10.5 heart RT-PCR of various X*^UtxGT2Δ^* and Y*^UtyGT^* allelic combinations. A moderate, but not significant downregulation of ANF was observed in X*^Utx−^* Y*^Uty+^* hearts (76% of WT, t-test p-value = 0.06), but was significantly compromised in X*^Utx−^* X*^Utx−^* (52% of WT, t-test p-value relative to X*^Utx+^* Y*^Uty+^* = 0.005) and X*^Utx−^* Y*^Uty−^* (57% of WT, t-test p-value relative to X*^Utx+^* Y*^Uty+^* = 0.02, N>4 per genotype) hearts.

It has been documented that UTX can associate with heart transcription factors and with the SWI/SNF chromatin remodeler, BRG1 [39]. It has been hypothesized that UTX association with these factors mediates H3K27 demethylase dependent and demethylase independent induction of the cardiomyocyte specification program. As UTX and UTY have redundant demethylase independent function in embryonic development, we examined whether UTY can also associate with these proteins. Co-transfection of Myc-UTY with Flag-BRG1 followed by immunoprecipitation demonstrated that UTY associates with BRG1 (Figure 8A). Myc-UTY also co-immunoprecipitated with Flag-NKX2–5, Flag-TBX5, and Flag-SRF (Figure 8B and Figure S10A). Thus, UTY can form the same protein complexes as UTX with respect to BRG1 and heart transcription factors.

To examine function of UTY in directing activation of downstream heart transcription factor targets, we assessed the regulation of one previously characterized target, atrial natriuretic factor (ANF) [39]. Co-transfection of NKX2–5 with a ANF promoter-Luciferase reporter construct demonstrated a significant upregulation in expression off the ANF promoter (Figure 8C). The reporter expression was significantly enhanced when NKX2–5 was co-transfected with UTY (Figure 8C). The level of ANF reporter transcriptional enhancement was relatively weaker with UTY as compared to UTX, but this is most likely due to a reduction in the transfection efficiency of full-length UTY relative to UTX (as demonstrated in Figure 7A and 7B). UTY also significantly enhanced the ANF reporter response to TBX5 (Figure S10B). Finally, ANF expression was significantly affected in the hearts of only X*^Utx−^* X*^Utx−^* and X*^Utx−^* Y*^Uty−^* embryos (with 52% and 57% level of expression respective to X*^Utx+^* Y*^Uty+^* controls, Figure 8D). X*^Utx−^* Y*^Uty+^* hemizygotes only had a moderate loss of ANF expression (76% expression level respective to X*^Utx+^* Y*^Uty+^*) that was not statistically significant from wild type controls due to the variability in ANF expression. In summary, both UTX and UTY can associate with heart transcription factors to modulate expression of downstream targets.

## Discussion

We have undertaken a rigorous genetic analysis contrasting UTX and UTY function in mouse embryonic development. In alignment with current literature, *Utx* homozygous females are lethal in mid-gestation with a block in cardiac development [39]. We now demonstrate that *Utx* hemizygous mutant males are viable at late embryonic timepoints in expected Mendelian frequencies. In fact, approximately 25% are capable of reaching adulthood. Our comprehensive phenotypic analysis of *Utx* hemizygous males illustrates that these embryos are phenotypically normal at mid-gestation and lack the cardiovascular dysfunction of *Utx* homozygous females. This stark phenotypic disparity suggests that UTY may compensate for the loss of UTX in the male embryo. Compound hemizygous *Utx*/*Uty* mutant male embryos phenocopy the cardiovascular and gross developmental delay of homozygous females, proving that UTX and UTY have redundant function in embryonic development. As we have demonstrated that mouse UTY lacks H3K27 demethylase activity *in vivo*, the overlap in embryonic UTX and UTY function is due to H3K27 demethylase independent activity. Given the widespread developmental delay and pleiotropy, it is difficult to assess the primary defect and tissue(s) responsible for UTX and UTY redundancy. The presence of functional UTY in *Utx* hemizygous males is not capable of preventing peri-natal runting and lethality, suggesting that UTX and UTY are not completely overlapping in activity. These later phenotypes could be due to H3K27 demethylase dependent activity of UTX. Furthermore, the lack of phenotype in *Uty* hemizygotes demonstrates the absence of any essential UTY specific function in mouse development.

The UTY Jumonji-C domain has maintained high conservation in the absence of catalytic H3K27 demethylase activity. JMJD3 mediated regulation of lymphocyte Th1 response requires an intact Jumonji-C domain, but is also not dependent on H3K27 demethylation [37]. Therefore, this domain may be an essential structural protein component, a protein binding domain, or a domain that may demethylate non-histone substrates. UTX and UTY can associate in a common protein complex and can both interact with RBBP5 of the H3K4 methyl-transferase complex. UTX, UTY, and JMJD3 all associate with H3K4 methyl-transferase complexes from multiple mouse and human cell types [23], [25], [44], [45]. The *Fnbp1* promoter is bound by UTX, and gene expression is positively regulated by both UTX and UTY in MEFs. Based on our histone profiling at this locus, UTX and UTY affect the deposition of H3K4 methylation, not H3K27me3 demethylation. Therefore, the common UTX/UTY pathway in embryonic development may involve gene activation rather than removal of gene repression. JMJD3 has been linked more directly to transcriptional activation as the protein complexes with and facilitates factors involved in transcriptional elongation [46]. One cardiac target of UTX regulation, atrial natriuretic factor (ANF), was misregulated in ES cell differentiation [39]. Cell culture experiments suggest that ANF may be a target of both H3K27 demethylase dependent and demethylase independent regulation; however, this study could not distinguish UTX versus UTY function in ES cell differentiation. Both UTX and UTY affect the transcriptional response of an exogenous ANF reporter in the presence of heart specific transcription factors, suggesting that UTX and UTY can operate more directly by aiding in transcriptional activation of this gene rather than altering chromatin structure. Consistently, ANF expression was affected in X*^Utx−^* X*^Utx−^* and X*^Utx−^* Y*^Uty−^* embryonic hearts. UTX and UTY can both associate with the SWI/SNF chromatin remodeler BRG1, which has been hypothesized to mediate histone demethylase independent gene regulation, but the relevance and mechanism of this interaction is not known. Drosophila UTX associates with BRM (orthologous to BRG1) and CBP (a H3K27 acetyl-transferase), and the coupling of H3K27 demethylation with H3K27 acetylation may be essential for switching from a silent to active state [47].

Female cells are subject to gene silencing of one X-chromosome (X-inactivation) to balance gene dosage with males. Theory on establishing X-inactivation for X-Y chromosome homologs hypothesizes that the initial entry step is loss of function or expression of the Y homolog to create dosage imbalance [38]. This prediction also dictates that conservation of X-Y homolog function will maintain gene dosage between sexes, and the female X-homolog will not experience pressure to inactivate. *Utx* and *Uty* represent a unique paradox to this untested theory; UTY has lost demethylation activity yet *Utx* escapes X-inactivation. We now demonstrate that UTX and UTY have retained embryonic redundancy, verifying the presumed correlations between X-inactivation escape and functional dosage balance. *Zfx*, *Sox3*, and *Amelx* represent unbalanced X-chromosome genes; they have similar hemizygous and homozygous mutant phenotypes indicating that the Y chromosome homologs have lost redundant function [48], [49], [50], [51], [52], [53], [54]. *Zfx* and *Sox3* are inactivated, while the *Amelx* inactivation status is unknown [55], [56]. Of all mouse X and Y chromosome homologs, only *Utx*, *Kdm5c*, and *Eif2s3x* are known to escape X-chromosome inactivation [28], [56], [57], [58]. Interestingly, both KDM5C (SMCX) and its Y chromosome homolog, KDM5D (SMCY) have retained catalytic activity in demethylation of H3K4 di and tri-methyl residues [59], [60], [61], [62]. In contrast to *Utx* X-chromosome escape driven by demethylation independent redundancy, *Kdm5c* may escape inactivation due to demethylation dependent redundancy. Our study is the first to demonstrate that an X-Y homologous pair that escapes X inactivation maintains functional conservation, and this escape may stem from an evolutionary benefit to maintain UTY demethylation independent function.

H3K27 demethylases are hypothesized to function in early developmental activation of “bivalent” PRC2 targets by coordinating H3K27 demethylation with H3K4 methylation. The H3K27 demethylation dependent phenotype (UTX specific) of *Utx* hemizygotes is not apparent until birth. The UTX H3K27 demethylase activity is dispensable for function in *C. elegans* [63]. Remarkably, the mammalian embryo, having numerous examples of H3K27me3 repression in early development, can survive to term without UTX histone demethylation. It is possible that there is further redundancy between UTX and JMJD3. JMJD3 mutant mice are not well characterized, but have been reported to be peri-natal lethal with distinct features in comparison to *Utx* hemizygotes [32]. Therefore, it is likely that JMJD3 has distinct targets in development. Overall, the earliest H3K27 demethylation dependent phenotypes for all members of this gene family do not manifest until late embryonic development. This timepoint is much later than the converse early embryonic phenotypes from mutations in the H3K27 methyl-transferase complex [7], . Thus, there appears to be a lack of interplay between H3K27 methylation and demethylation in gene regulation, and the early embryonic removal of H3K27me3 from PRC2 mediated processes (such as ES cell differentiation, reactivation of the inactive X-chromosome, or establishing autosomal imprinting) may involve other mechanisms such as histone turnover or chromatin remodeling. H3K27 demethylases may certainly have crucial roles in the specification of progenitor cell populations of organ systems essential in peri-natal or postnatal viability, and genetic model systems will best assess the functional impact that H3K27 demethylation plays in these processes.

## Methods

### Cell culture and constructs

HEK293T were maintained in DMEM supplemented with Glutamine, Pen-Strep, and 10%FBS. Flag-Human UTX (Plasmid #17438) and UTY (Plasmid #17439) were obtained through Addgene [29]. The N-terminus of H-UTX and H-UTY were deleted with QuikChange Lightning (Agilent) as directed producing H-UTX C-terminus 880–1401 (Genbank: NP_066963.2) and H-UTY C-terminus 827–1343 (Genbank: NP_009056.3, an N-terminal His tag was also incorporated into both constructs). Site directed mutagenesis was performed via QuikChange Lightning (Agilent) as directed to produce point mutations. The mouse UTX C-terminus (880–1401) deviated from Human UTX at 2 residues, R1073K and S1263N (According to the Sanger Vega server, the primary Utx transcript Kdm6a-001 encodes for Genbank: CAM27157, we also detected this transcript in E14 ES cell RT-PCR). These changes were created in the H-UTX C-terminus to generate the M-UTX construct. The Flag-tagged mouse UTY C-terminus (692–1212, Genbank: NP_033510.2) was subcloned by RT-PCR of E14 ES cell RNA and introduced into the same vector as the other UTX constructs (PCS2+MT backbone). HA tagged H-UTX was obtained through Addgene [24]. Flag tagged mouse JMJD3 was generously provided by Burgold et al. [27]. Flag tagged BRG1 was obtained through Addgene (Plasmid #19143). Flag tagged NKX2–5 (Plasmid #32969), TBX5 (Plasmid #32968), and SRF (Plasmid #32971) were obtained through Addgene and recombined into DEST26 (Invitrogen). Flag tagged NKX2–5 was also generously provided by Benoit Bruneau [64].

### Transfections, immunofluorescence, Western blotting, antibodies

Transfection of HEK293T was accomplished with Lipofectamine 2000 as directed (Invitrogen). Lipid complexes were removed 24 hours post-transfection, and analysis was performed after 48 hours total. Fixation, extraction, and immunofluorescence were performed as described [65]. Immunofluorescence antibodies include anti-Flag (Sigma F3165, 1∶500), anti-H3K27me3 (Millipore 07-449, 1∶500), anti-H3K27me2 (Millipore 07-452, 1∶500), and anti-H3K4me2 (Millipore 07-030, 1∶500). Cells were imaged with Zeiss axiovision software. Image stacks were deconvolved and z-projected. Quantification of H3K27me3 immunofluorescence was performed on deconvolved z-projected stacks (with no pixel saturation in images) using ImageJ software (NIH). The average mean H3K27me3 signal was calculated for untransfected and transfected cells in a given image. For each image, the relative % H3K27me3 was determined, and the average relative % H3K27me3 was calculated for >15 images per construct. For western blotting, nuclear lysates were prepared according to Invitrogen's nuclear extraction protocol. Immunoprecipitations were carried out with 50 µl Flag beads (Sigma A2220) in buffer A as described, using 500 µg (UTY-UTX, RBBP5, BRG1, TBX5, SRF associations) or 1 mg (UTY-NKX2–5 association) of lysate [44]. Immunoprecipitation reactions were boiled off beads and run with 10% input on an 8% SDS-PAGE gel. Histone extractions were prepared as described [66]. Western blotting was performed as described [67] with anti-Flag (Cell Signaling 2368, 1∶4000), anti-RBBP5 (Bethyl Labs A300-109A, 1∶5000), anti-HA (Roche 11867423001, 1∶10000), anti-Myc (Abcam ab9132, 1∶5000), anti-H3K27me3 (Millipore 07-449, 1∶2000), anti-H3 (Millipore 06-755, 1∶5000), and anti-UTX [24].

### MEF generation, RT–PCR, and ChIP

E10.5 and E12.5 MEFs were generated by removal of the head and interior organs of respective embryos. The remaining body was passed through a 20G needle 6× and plated in DMEM supplemented with Glutamine, Pen-Strep, and 15%FBS. After 3 passages, RNA was isolated with Trizol, and cleaned with an RNeasy kit (Quiagen). RNA from 3 distinct WT X*^Utx+^* Y*^Uty+^* MEF lines was compared to 3 X*^UtxGT2Δ^* X*^UtxGT2Δ^* lines on an Illumina bead array (University of Tennessee Health Science Center). All genes significantly decreased in X*^UtxGT2Δ^* X*^UtxGT2Δ^* MEFs were cross-referenced to the list of UTX bound promoters in human fibroblasts [30]. These genes were analyzed by qRT-PCR (Bio-Rad SsoFast EvaGreen, CFX96 real time system) in all mutant MEF combinations to identify UTY regulated genes. ChIP was performed on these MEFs according to Rahl et al. [68]. MEFs (5×10^6^ cells) were sonicated by a Branson Sonifier at 15% duty cycle (0.7 s on 0.3 s off). ChIP was performed with anti-H3K27me3 (Millipore 07-449, 10 µl), anti-H3K4me3 (Abcam ab1012, 5 µl), anti-Myc (Abcam ab9132, 10 µl), anti-UTX (Santa Cruz H-300, 50 µl), or Rabbit IgG (Sigma, I5006) and qPCR was performed as described above.

### Luciferase assay

We received the ANF promoter-Luciferase reporter construct from Benoit Bruneau [69]. This construct was co-transfected in the presence of NKX2–5 or TBX5 with or without UTX or UTY. Luciferase activity was measured using the Promega Dual Luciferase Reporter Assay System on the Promega Glomax Multi Detection System. All readings were normalized to a Renilla Luciferase control that was co-transfected with all samples.

### Mouse strains

*Kdm6a^Gt(RRA094)Byg^* (X*^UtxGT1^*), *Kdm6a^tm1a(EUCOMM)Wtsi^* (X*^UtxGT2fl^*), and *Uty^Gt(XS0378)Wtsi^* (Y*^UtyGT^*) ES cells were obtained from BayGenomics (through MMRRC), EUCOMM, and SIGTR (through MMRRC) respectively. All ES cells were injected into C57BL/6J host blastocysts for chimera generation. Chimeras were crossed to CD1 to assess germline transmission, and were maintained on either a mixed CD1 background or were backcrossed to 129/SvJ or C57BL/6J. *Sox2Cre* and *Rosa^Flp^* transgenes were obtained from The Jackson Laboratory [40]. The *VasaCre* transgene was developed by Gallardo et al. [70].

### Mouse crosses

All mouse experimental procedures were approved by the University of North Carolina Institutional Animal Care and Use Committee. *Utx* homozygous data was generated either by crosses between *Utx* hemizygous males and *Utx* heterozygous females, or by crosses between X*^UtxGT2fl^* Y*^Uty+^ VasaCre* males and X*^Utx+^* X*^UtxGT2Δ^* heterozygous females. X*^UtxGT2fl^* Y*^Uty+^ VasaCre* males were utilized because of an initial difficulty in generating X*^UtxGT2Δ^* Y*^Uty+^* males and due to the efficient and specific activity of *VasaCre* in the male germline [70]. *Utx* hemizygous phenotypic data was developed from the previously mentioned homozygous crosses or through crosses between a WT male and heterozygous *Utx* female. Compound hemizygous *Utx*/*Uty* embryos were generated by crossing heterozygous *Utx* females with hemizygous *Uty* males. Embryos were PCR genotyped from yolk sac samples for *Utx* and were sexed by a PCR genotyping scheme to distinguish *Utx* from *Uty*. All primer sequences are available upon request.

### Histology, *in situ* hybridization, and LacZ staining

Histology samples, *in situ* hybridization, and LacZ staining were performed as described [71]. *In situ* hybridization probes were generated to be identical to previous literature [72].

## Supporting Information

Figure S1 Schematic of genotyping strategies for *Utx* and *Uty* alleles. (A) The X*^UtxGT1^* allele was genotyped with a three-primer scheme spanning the insertion site in intron 3. (B) The X*^UtxGT2fl^* allele was verified by Southern blotting with an HpaI restriction digest. HpaI sites are noted as “H”, and the 5′ probe location is marked as a red box. The introduction of a novel HpaI site within the targeting cassette reduces the HpaI product from 17-Kb to 10-Kb. A three-primer scheme was designed for genotyping. Due to a deletion of intron 3 within the targeting vector, the product size of primers 1-2 will be larger in WT than in X*^UtxGT2fl^*, even with the introduction of the loxP site. Primers 3-2 will only amplify if Cre recombination takes place to delete exon 3. (C) The Y*^UtyGT^* insertion site was not mapped because intron 4 is approximately 25-Kb. The allele was verified by a RT-PCR three-primer genotyping scheme.
(TIF)

Figure S2 Verification of *Utx* and *Uty* alleles. (A–C) Verification of the X*^UtxGT1^* allele. (A) Trap specific primers between *Utx* exon 2 and the B-Gal reporter amplify the expected band in X*^UtxGT1^* Y*^+^* ES cells. WT male E14 ES cells were used as a control. (B) The gene trap DNA location was mapped within *Utx* intron 3, and primers were designed to distinguish wild type (WT) and gene trap (GT) alleles in mice generated from these cells. (C) Quantitative RT-PCR downstream of the gene trap (exons 23–25) from tail RNA of X*^UtxGT1^* Y*^+^* mice demonstrate the gene trap effectiveness. (D–F) Verification of the X*^UtxGT2fl^* allele. (D) Southern blotting of WT and X*^UtxGT2fl^* Y^+^ ES cells using a 5′ probe and HpaI digest demonstrated the expected shift in banding due to a novel restriction site. (E) A PCR genotyping scheme was designed to distinguish WT (X^+^), X*^UtxGT2Δ^*, and X*^UtxGT2fl^* alleles in mice produced from these ES cells. (F) Quantitative RT-PCR downstream of the gene trap (exons 23–25) from tail RNA of X^UtxGT2fl^ Y^+^ mice demonstrate the gene trap effectiveness. (G) Verification of the Y*^UtyGT^* allele. A RT-PCR genotyping scheme was designed to distinguish WT and Y*^UtyGT^* alleles in ES cells.
(TIF)

Figure S3 *Utx* and *Uty* have similar expression patterns. (A) Whole mount B-galactosidase reporter assay on X*^Utx+^* X*^UtxGT1^* (A-ii, iii) and X*^UtxGT1^* X*^UtxGT1^* (A-iv) E10.5 embryos. Embryos were cleared in A-iii, iv. (B) *In situ* hybridization of *Utx* sense control (B-i, iv), *Utx* antisense (B-ii, v), and *Uty* antisense (B-iii, vi) probes on E10.5 sagittal sections of WT male embryos.
(TIF)

Figure S4 Mouse UTY and corresponding mutation of the UTX catalytic domain abolish H3K27me3 demethylation. (A) Western blot of transfections from the H3K27me3 demethylase assay in Figure 6. Flag tagged UTX and UTY constructs are expressed at similar levels in this assay, Rbbp5 blotting served as a loading control. (B) Quantification of H3K27me3 immunofluorescence assay from Figure 5. In a given image, the average H3K27me3 immunofluorescence for transfected and untransfected cells was quantified. The average of the % H3K27me3 immunofluorescence relative to untransfected cells was graphed (N>15 images per transfection).
(TIF)

Figure S5 Mouse UTY has no H3K27me2 demethylase activity. (A) HEK293T cells were transfected with Flag tagged C-terminal human (H) and mouse (M) UTX and UTY constructs. Transfected cells (white arrows) over-expressing H-UTX and M-UTX (green channel) exhibited global loss of H3K27me2 immunofluorescence (red, top 2 panels). H-UTX Y1135C and M-UTY had no loss of H3K27me2 (bottom 2 panels). (B) Expression of WT H-UTX had no effect on H3K4me2.
(TIF)

Figure S6 Alignment of human and mouse UTX, UTY, and JMJD3. Alignment of the C-terminal 880–1401 amino acids of H-UTX and corresponding regions of human and mouse UTX, UTY, and JMJD3. The JmjC domain is boxed in pink. Several residues in H-UTX predicted to be important for H3K27 demethylation are mutated in mouse or human UTY. These residues are boxed in black, and these point mutations were made in H-UTX (listed above the box) or JMJD3 (listed below the box).
(TIF)

Figure S7 Alignment of the JmjC domain of UTX, UTY, and JMJD3. JmjC domain sequences were aligned from all identified homologs of UTX, UTY, and JMJD3. All species have UTX residue H1146 and E1148 required for Iron binding in the demethylase reaction. Y1135 crucial for H3K27me3 binding and T1143 essential for ketoglutarate binding in the demethylase reaction are conserved throughout all species except for mouse UTY.
(TIF)

Figure S8 Alignment of the JmjC domain of KDM6, KDM2, KDM7, and KDM3. JmjC domain sequences were aligned from human, mouse, a non-mammalian vertebrate (if protein sequences were available), and an invertebrate (if protein sequences were available) species for identified KDM6, KDM2, KDM7, and KDM3 family members. The UTX T1143 essential for ketoglutarate binding in the demethylase reaction is conserved throughout all species except for mouse UTY.
(TIF)

Figure S9 UTX mutant MEFs have unaltered levels of H3K27me3 and *FNBP1* is bound by UTX and UTY. (A) Western blot of H3K27me3 and total H3 following histone extraction from MEFs of the indicated genotypes. There is no change in the level of global H3K27me3 in lines with loss of UTX. (B) HEK293T cells were transfected with a Myc vector control, Myc-UTX or Myc-UTY. ChIP was performed with Myc antibody and qPCR tested association with a negative control (an intergenic region, grey bars), GAPDH (negative control, red bars), *FNBP1* (green bars), or HOXA9 (positive control, yellow bars). Myc-UTX and Myc-UTY associate with the *FNBP1* promoter. (C) ChIP was performed on primary MEFs with an IgG control or UTX antibody. ChIP with the UTX antibody was performed in wild-type X*^Utx+^* Y*^Uty+^* or X*^UtxGT2fl^* X*^UtxGT2fl^* MEFs and qPCR tested association with the *Fnbp1* promoter relative to a negative control intergenic region.
(TIF)

Figure S10 UTX and UTY associate with heart transcription factors and regulate expression of ANF. (A) Myc-UTY was co-transfected with the Flag negative control, Flag-TBX5, or Flag-SRF. Myc-UTY was co-immunoprecipitated by Flag-TBX5 and Flag-SRF. (B) ANF:Luciferase reporter assay. HEK293T were transfected with the reporter ANF:Luciferase construct alone (-), with TBX5, or with TBX5 and UTY. Reporter activity was significantly enhanced with the addition of UTY (t-test p-value = 0.004).
(TIF)

Table S1 *Utx* hemizygous genotype frequency on inbred backgrounds. Observed (Obs) and expected (Ex) frequencies of indicated genotypes (Geno) at embryonic (E) or postnatal (P) developmental stages with χ^2^ p-values (p-value) for the corresponding crosses to obtain each genotype. At E18.5 on the C57BL/6J background, 5 of the 8 observed X*^UtxGT1^* Y*^Uty+^* males were on the N8 generation.
(DOC)

Table S2 Genotype frequencies of Sox2Cre driven *Utx* mutation. Observed (Obs) and expected (Ex) frequencies of indicated genotypes (Geno) at embryonic (E) or postnatal (P) developmental stages with χ^2^ p-values (p-value) for the corresponding crosses to obtain each genotype.
(DOC)
