# Supplementary figures and images for: UTX and UTY Demonstrate Histone Demethylase-Independent Function in Mouse Embryonic Development
Source: PLoS Genet. 2012 Sep 27;8(9):e1002964. doi: 10.1371/journal.pgen.1002964 (PMC3459986; doi:10.1371/journal.pgen.1002964)

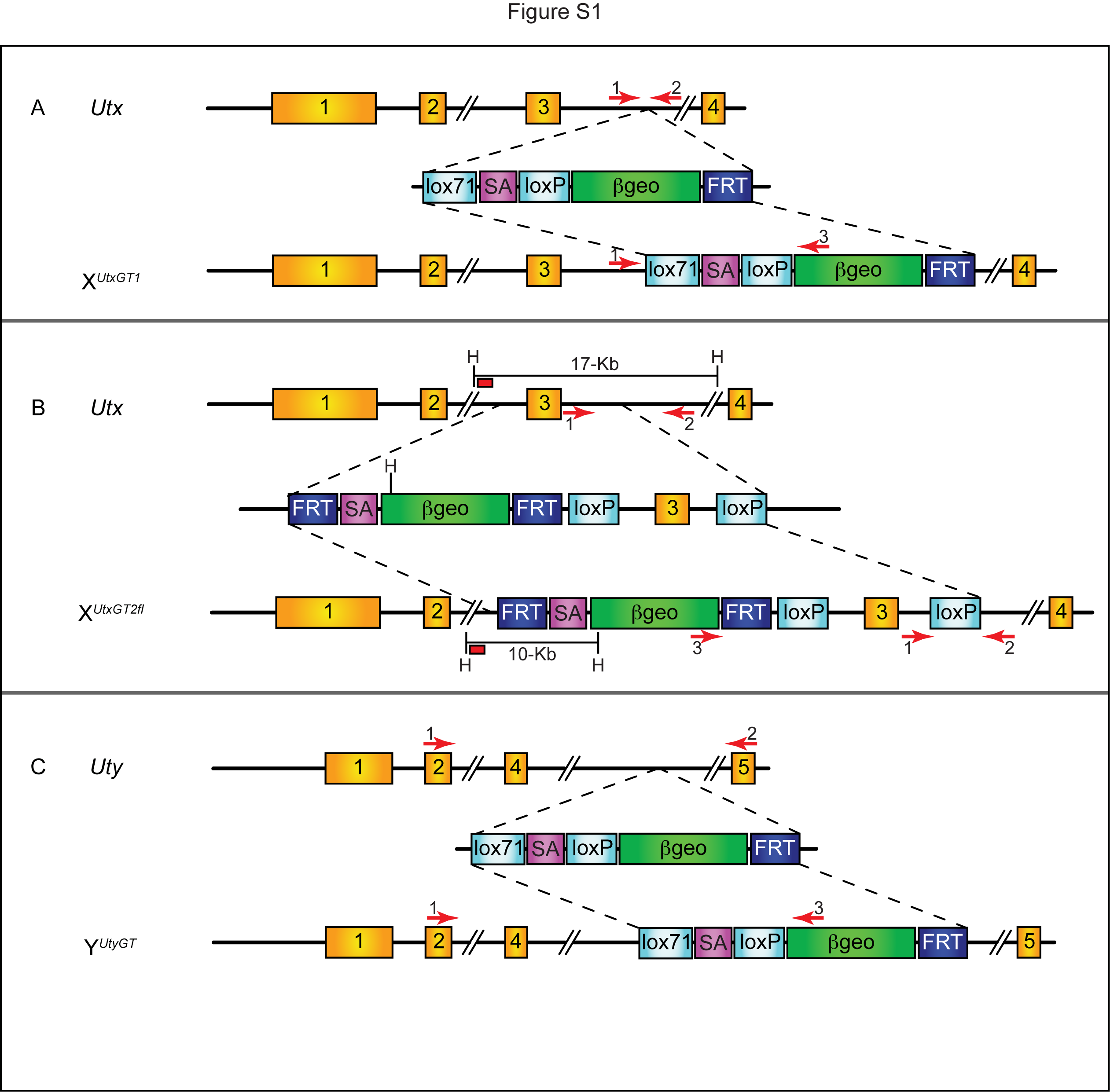

Supplement: Figure S1 — Schematic of genotyping strategies for Utx and Uty alleles. (A) The XUtxGT1 allele was genotyped with a three-primer scheme spanning the insertion site in intron 3. (B) The XUtxGT2fl allele was verified by Southern blotting with an HpaI restriction digest. HpaI sites are noted as “H”, and the 5′ probe location is marked as a red box. The introduction of a novel HpaI site within the targeting cassette reduces the HpaI product from 17-Kb to 10-Kb. A three-primer scheme was designed for genotyping. Due to a deletion of intron 3 within the targeting vector, the product size of primers 1-2 will be larger in WT than in XUtxGT2fl, even with the introduction of the loxP site. Primers 3-2 will only amplify if Cre recombination takes place to delete exon 3. (C) The YUtyGT insertion site was not mapped because intron 4 is approximately 25-Kb. The allele was verified by a RT-PCR three-primer genotyping scheme. (TIF) [file pgen.1002964.s001.tif]

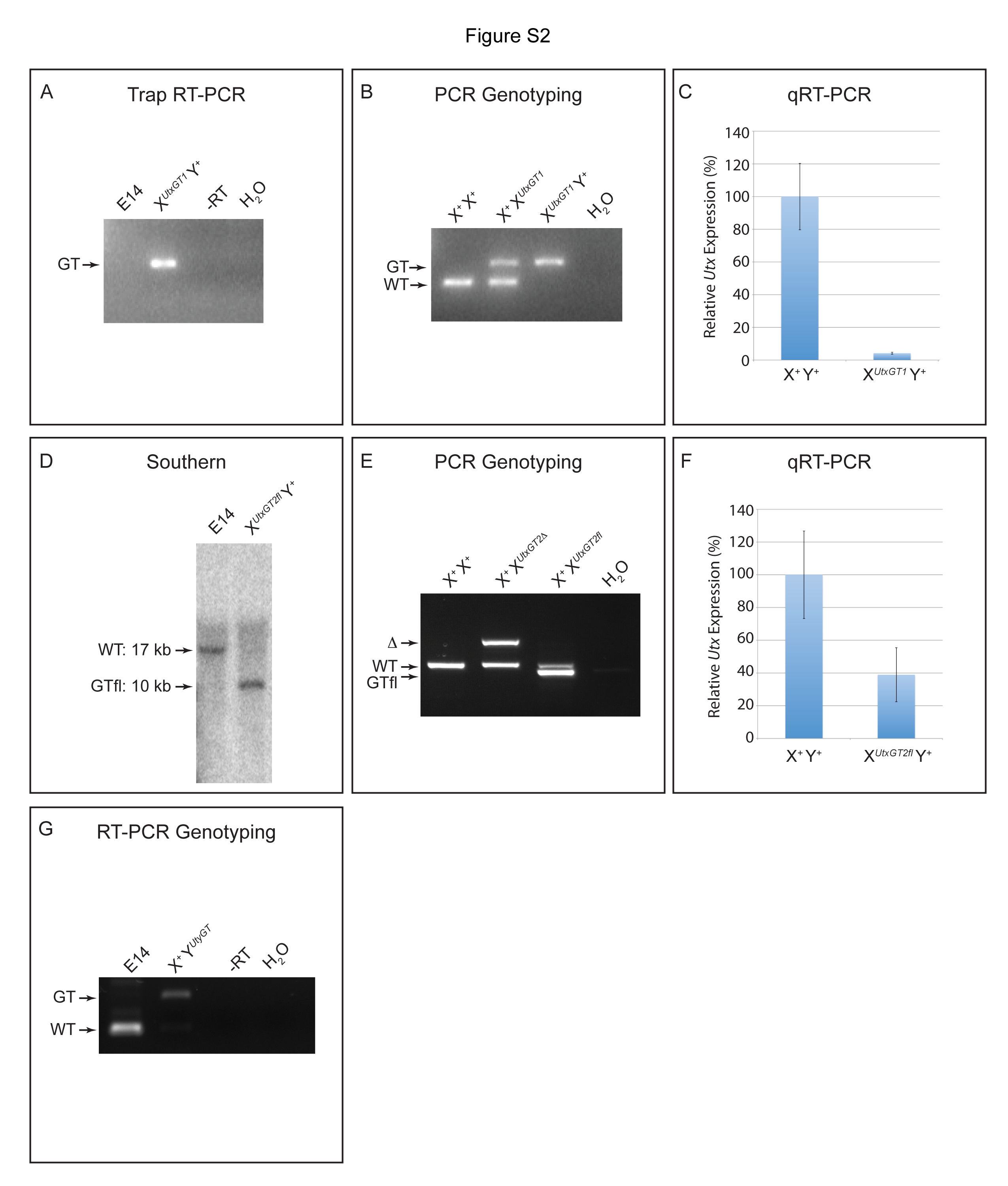

Supplement: Figure S2 — Verification of Utx and Uty alleles. (A–C) Verification of the XUtxGT1 allele. (A) Trap specific primers between Utx exon 2 and the B-Gal reporter amplify the expected band in XUtxGT1 Y+ ES cells. WT male E14 ES cells were used as a control. (B) The gene trap DNA location was mapped within Utx intron 3, and primers were designed to distinguish wild type (WT) and gene trap (GT) alleles in mice generated from these cells. (C) Quantitative RT-PCR downstream of the gene trap (exons 23–25) from tail RNA of XUtxGT1 Y+ mice demonstrate the gene trap effectiveness. (D–F) Verification of the XUtxGT2fl allele. (D) Southern blotting of WT and XUtxGT2fl Y+ ES cells using a 5′ probe and HpaI digest demonstrated the expected shift in banding due to a novel restriction site. (E) A PCR genotyping scheme was designed to distinguish WT (X+), XUtxGT2Δ, and XUtxGT2fl alleles in mice produced from these ES cells. (F) Quantitative RT-PCR downstream of the gene trap (exons 23–25) from tail RNA of XUtxGT2fl Y+ mice demonstrate the gene trap effectiveness. (G) Verification of the YUtyGT allele. A RT-PCR genotyping scheme was designed to distinguish WT and YUtyGT alleles in ES cells. (TIF) [file pgen.1002964.s002.tif]

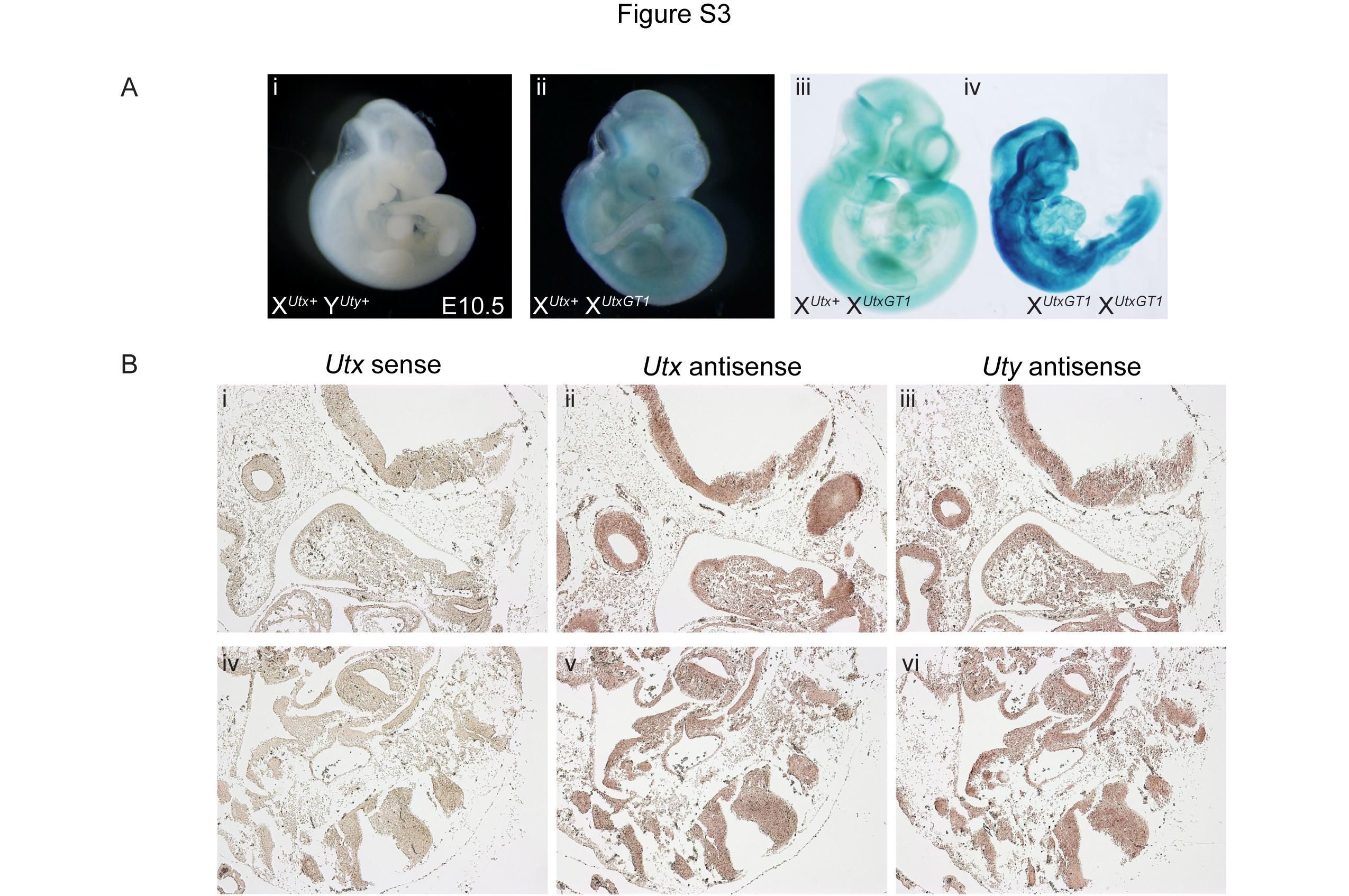

Supplement: Figure S3 — Utx and Uty have similar expression patterns. (A) Whole mount B-galactosidase reporter assay on XUtx+ XUtxGT1 (A-ii, iii) and XUtxGT1 XUtxGT1 (A-iv) E10.5 embryos. Embryos were cleared in A-iii, iv. (B) In situ hybridization of Utx sense control (B-i, iv), Utx antisense (B-ii, v), and Uty antisense (B-iii, vi) probes on E10.5 sagittal sections of WT male embryos. (TIF) [file pgen.1002964.s003.tif]

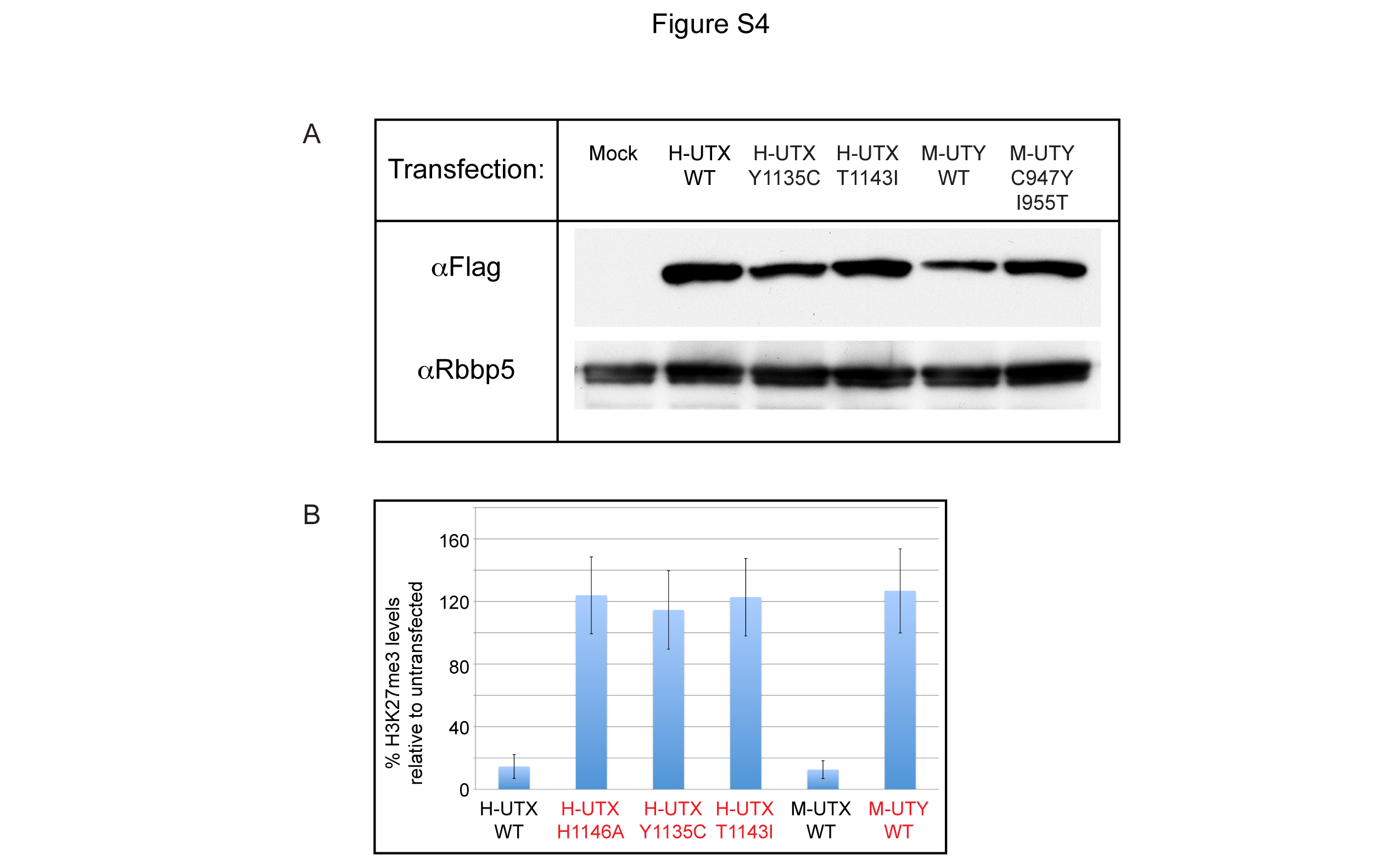

Supplement: Figure S4 — Mouse UTY and corresponding mutation of the UTX catalytic domain abolish H3K27me3 demethylation. (A) Western blot of transfections from the H3K27me3 demethylase assay in Figure 6. Flag tagged UTX and UTY constructs are expressed at similar levels in this assay, Rbbp5 blotting served as a loading control. (B) Quantification of H3K27me3 immunofluorescence assay from Figure 5. In a given image, the average H3K27me3 immunofluorescence for transfected and untransfected cells was quantified. The average of the % H3K27me3 immunofluorescence relative to untransfected cells was graphed (N>15 images per transfection). (TIF) [file pgen.1002964.s004.tif]

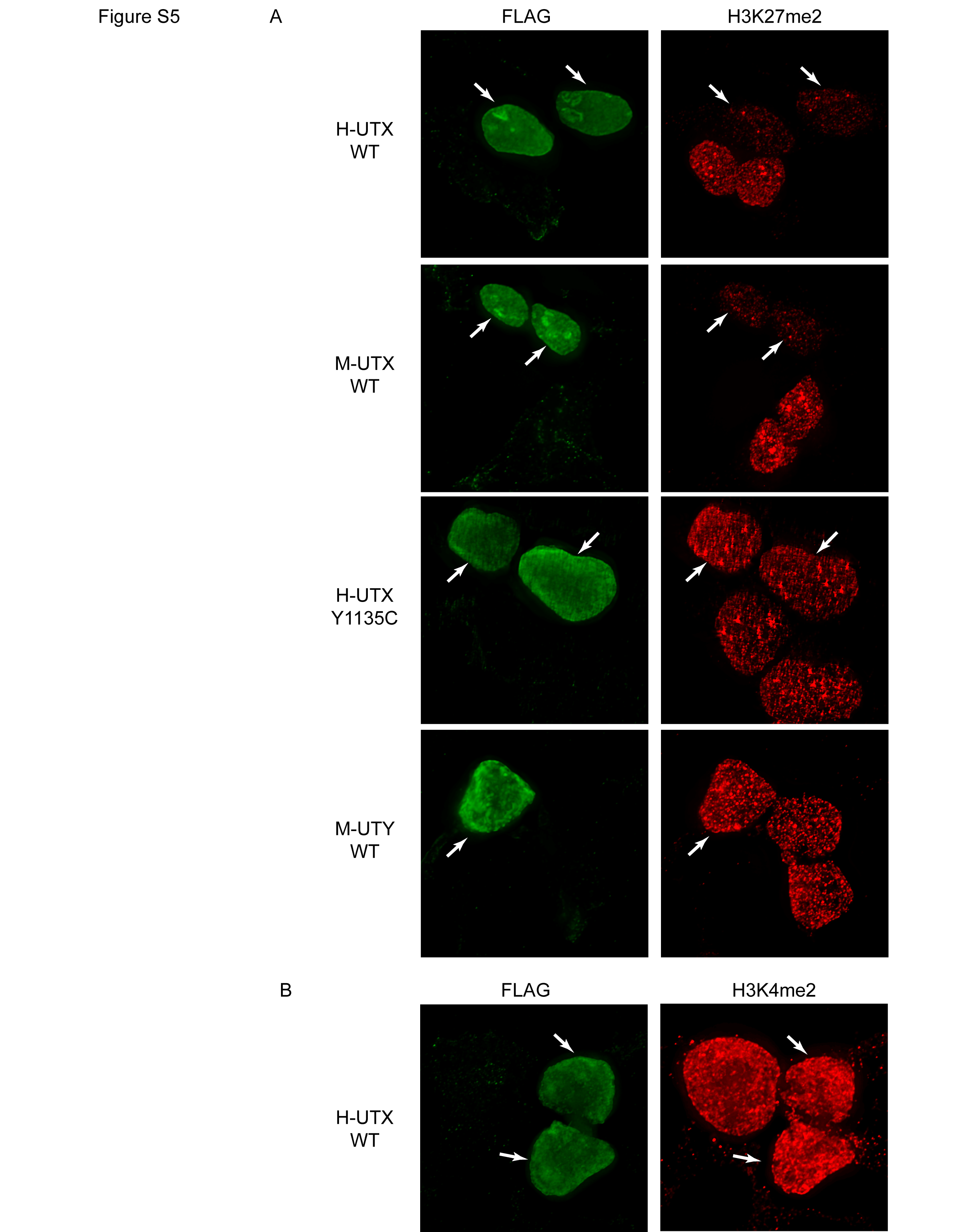

Supplement: Figure S5 — Mouse UTY has no H3K27me2 demethylase activity. (A) HEK293T cells were transfected with Flag tagged C-terminal human (H) and mouse (M) UTX and UTY constructs. Transfected cells (white arrows) over-expressing H-UTX and M-UTX (green channel) exhibited global loss of H3K27me2 immunofluorescence (red, top 2 panels). H-UTX Y1135C and M-UTY had no loss of H3K27me2 (bottom 2 panels). (B) Expression of WT H-UTX had no effect on H3K4me2. (TIF) [file pgen.1002964.s005.tif]

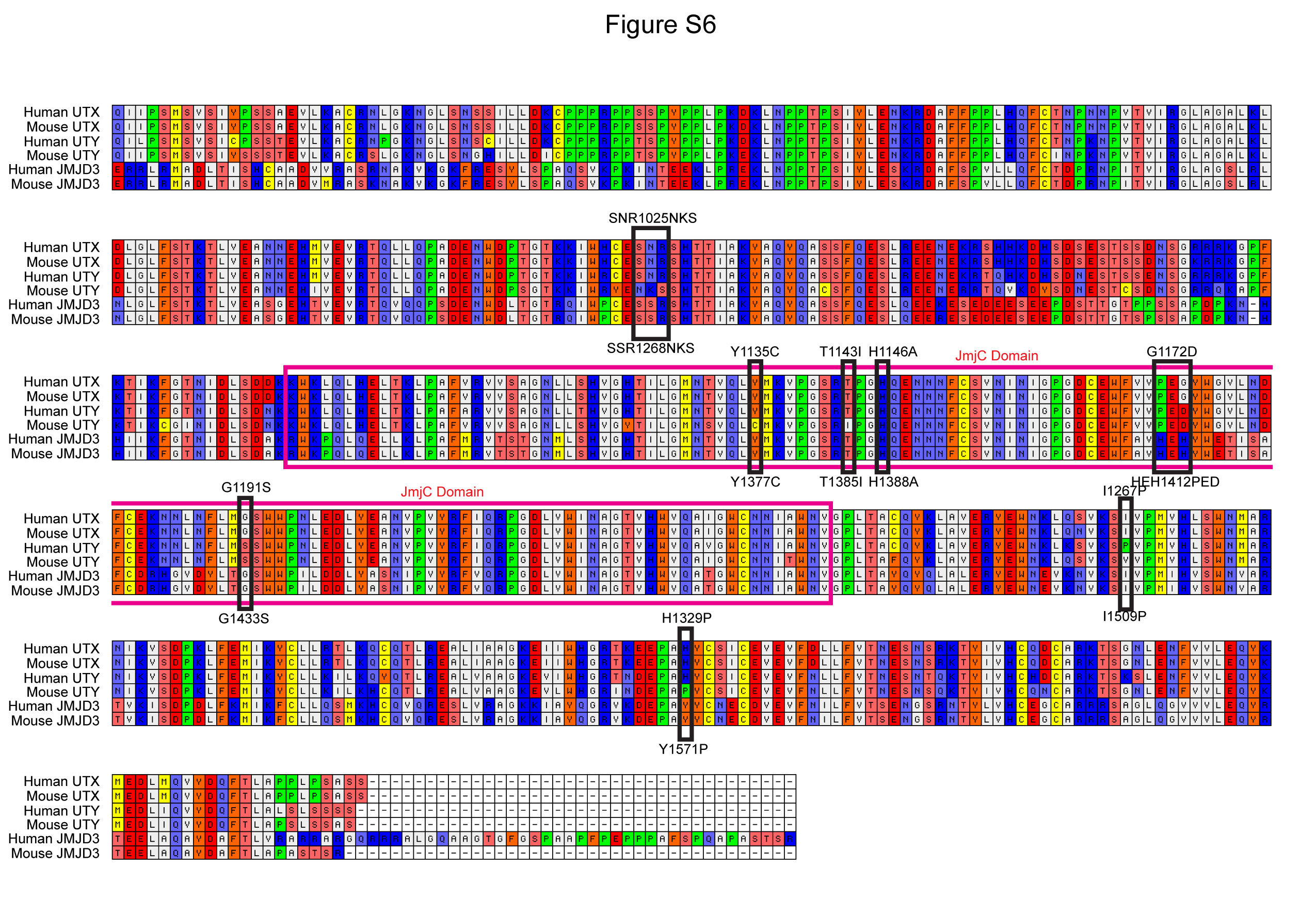

Supplement: Figure S6 — Alignment of human and mouse UTX, UTY, and JMJD3. Alignment of the C-terminal 880–1401 amino acids of H-UTX and corresponding regions of human and mouse UTX, UTY, and JMJD3. The JmjC domain is boxed in pink. Several residues in H-UTX predicted to be important for H3K27 demethylation are mutated in mouse or human UTY. These residues are boxed in black, and these point mutations were made in H-UTX (listed above the box) or JMJD3 (listed below the box). (TIF) [file pgen.1002964.s006.tif]

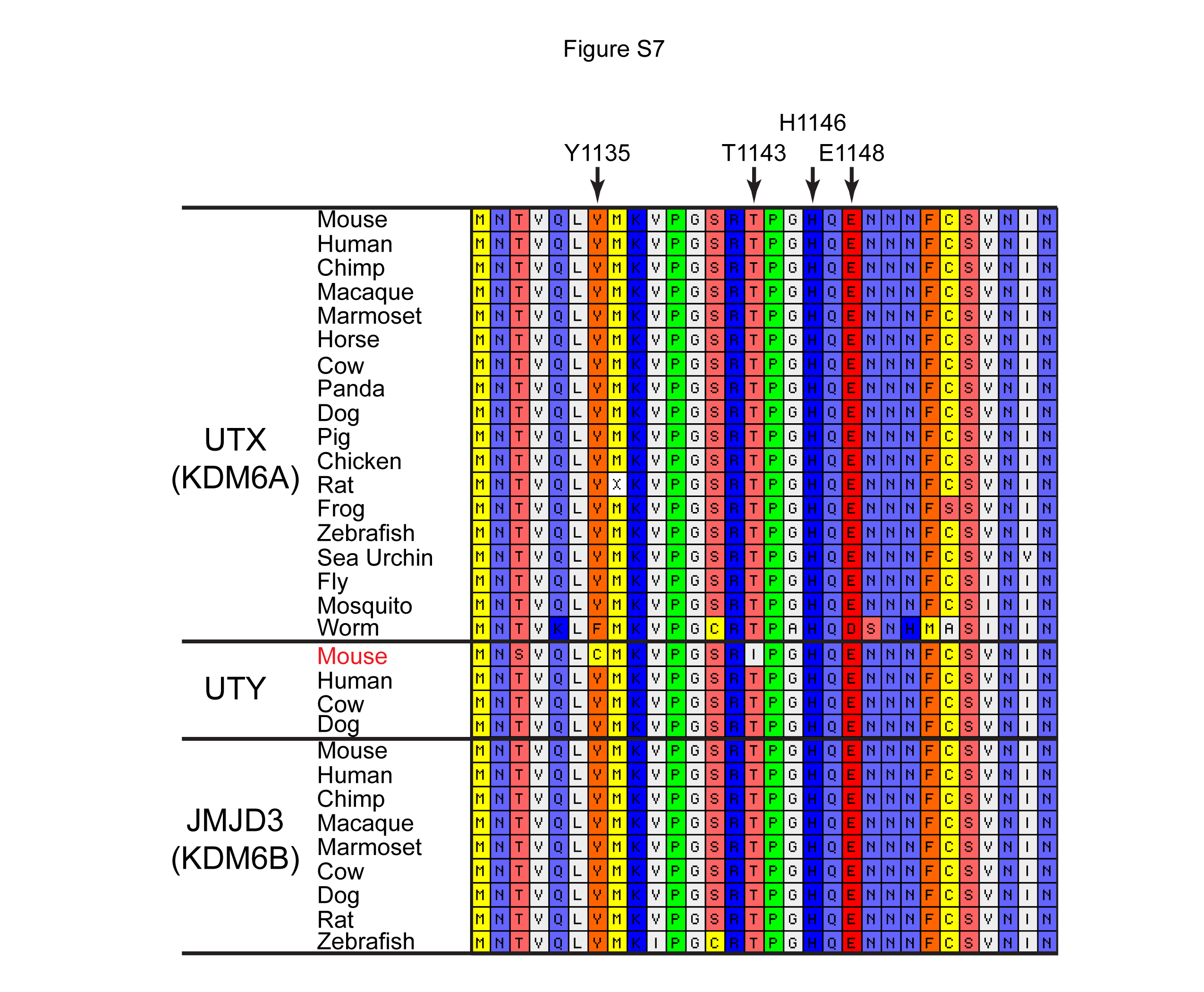

Supplement: Figure S7 — Alignment of the JmjC domain of UTX, UTY, and JMJD3. JmjC domain sequences were aligned from all identified homologs of UTX, UTY, and JMJD3. All species have UTX residue H1146 and E1148 required for Iron binding in the demethylase reaction. Y1135 crucial for H3K27me3 binding and T1143 essential for ketoglutarate binding in the demethylase reaction are conserved throughout all species except for mouse UTY. (TIF) [file pgen.1002964.s007.tif]

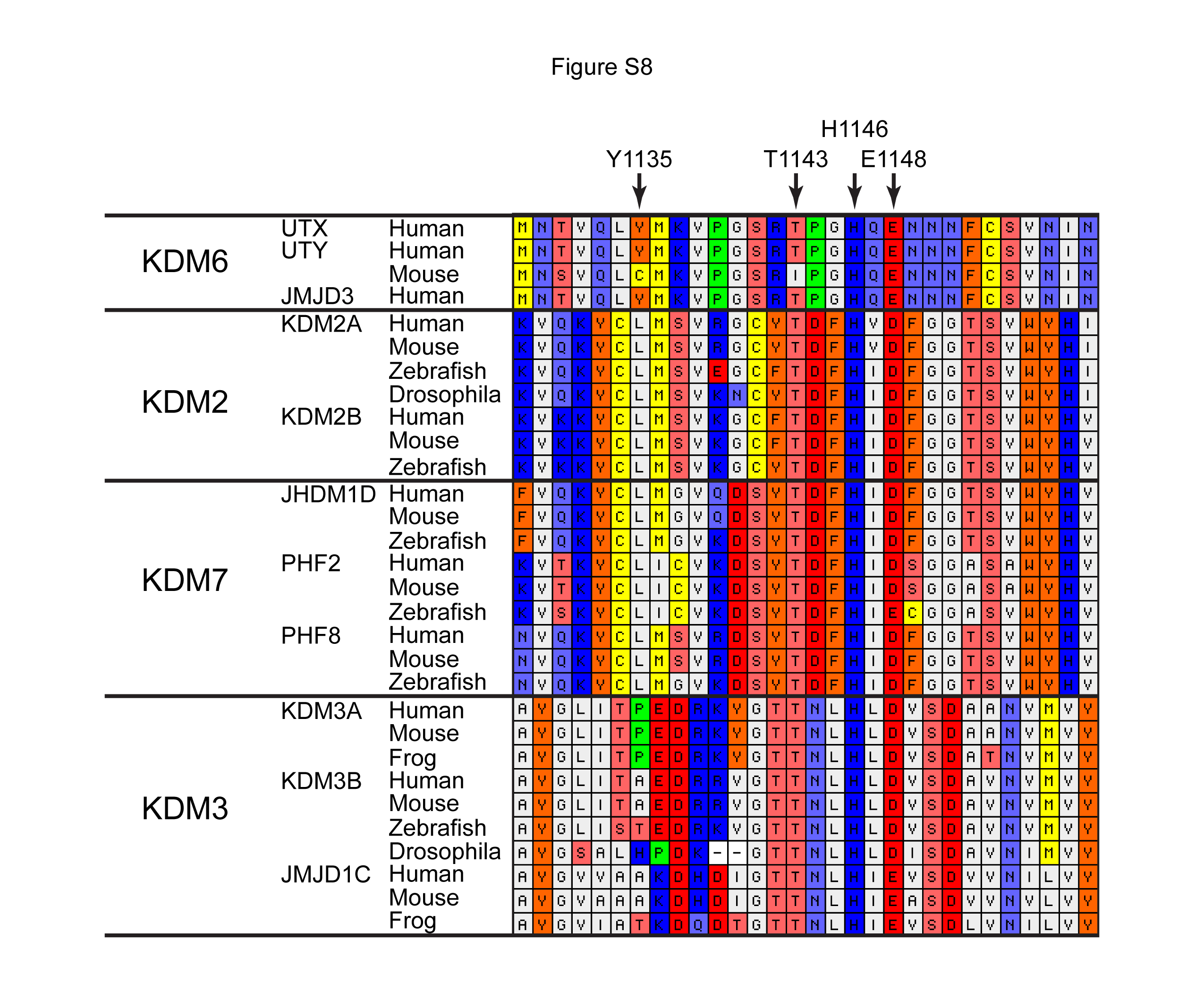

Supplement: Figure S8 — Alignment of the JmjC domain of KDM6, KDM2, KDM7, and KDM3. JmjC domain sequences were aligned from human, mouse, a non-mammalian vertebrate (if protein sequences were available), and an invertebrate (if protein sequences were available) species for identified KDM6, KDM2, KDM7, and KDM3 family members. The UTX T1143 essential for ketoglutarate binding in the demethylase reaction is conserved throughout all species except for mouse UTY. (TIF) [file pgen.1002964.s008.tif]

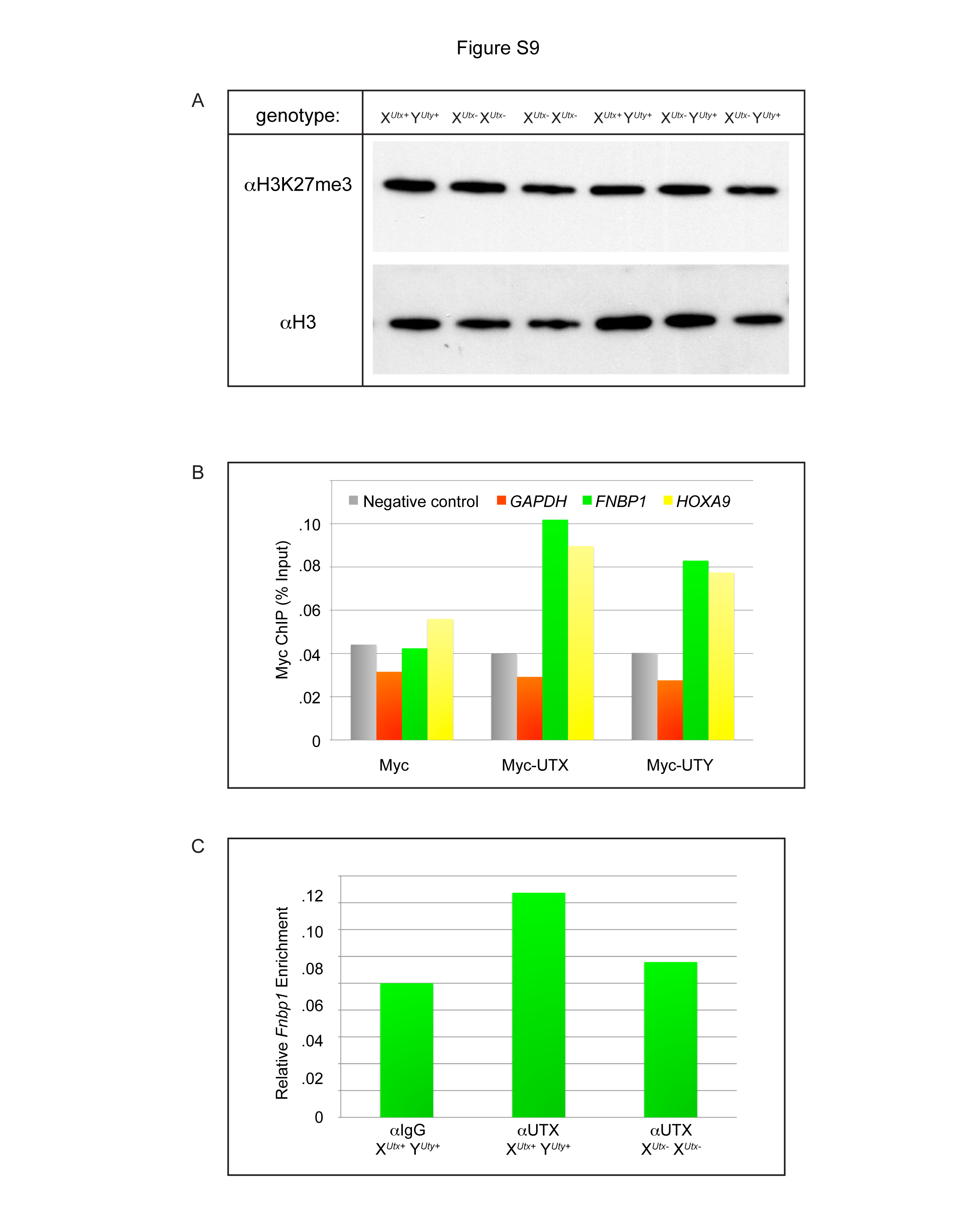

Supplement: Figure S9 — UTX mutant MEFs have unaltered levels of H3K27me3 and FNBP1 is bound by UTX and UTY. (A) Western blot of H3K27me3 and total H3 following histone extraction from MEFs of the indicated genotypes. There is no change in the level of global H3K27me3 in lines with loss of UTX. (B) HEK293T cells were transfected with a Myc vector control, Myc-UTX or Myc-UTY. ChIP was performed with Myc antibody and qPCR tested association with a negative control (an intergenic region, grey bars), GAPDH (negative control, red bars), FNBP1 (green bars), or HOXA9 (positive control, yellow bars). Myc-UTX and Myc-UTY associate with the FNBP1 promoter. (C) ChIP was performed on primary MEFs with an IgG control or UTX antibody. ChIP with the UTX antibody was performed in wild-type XUtx+ YUty+ or XUtxGT2fl XUtxGT2fl MEFs and qPCR tested association with the Fnbp1 promoter relative to a negative control intergenic region. (TIF) [file pgen.1002964.s009.tif]

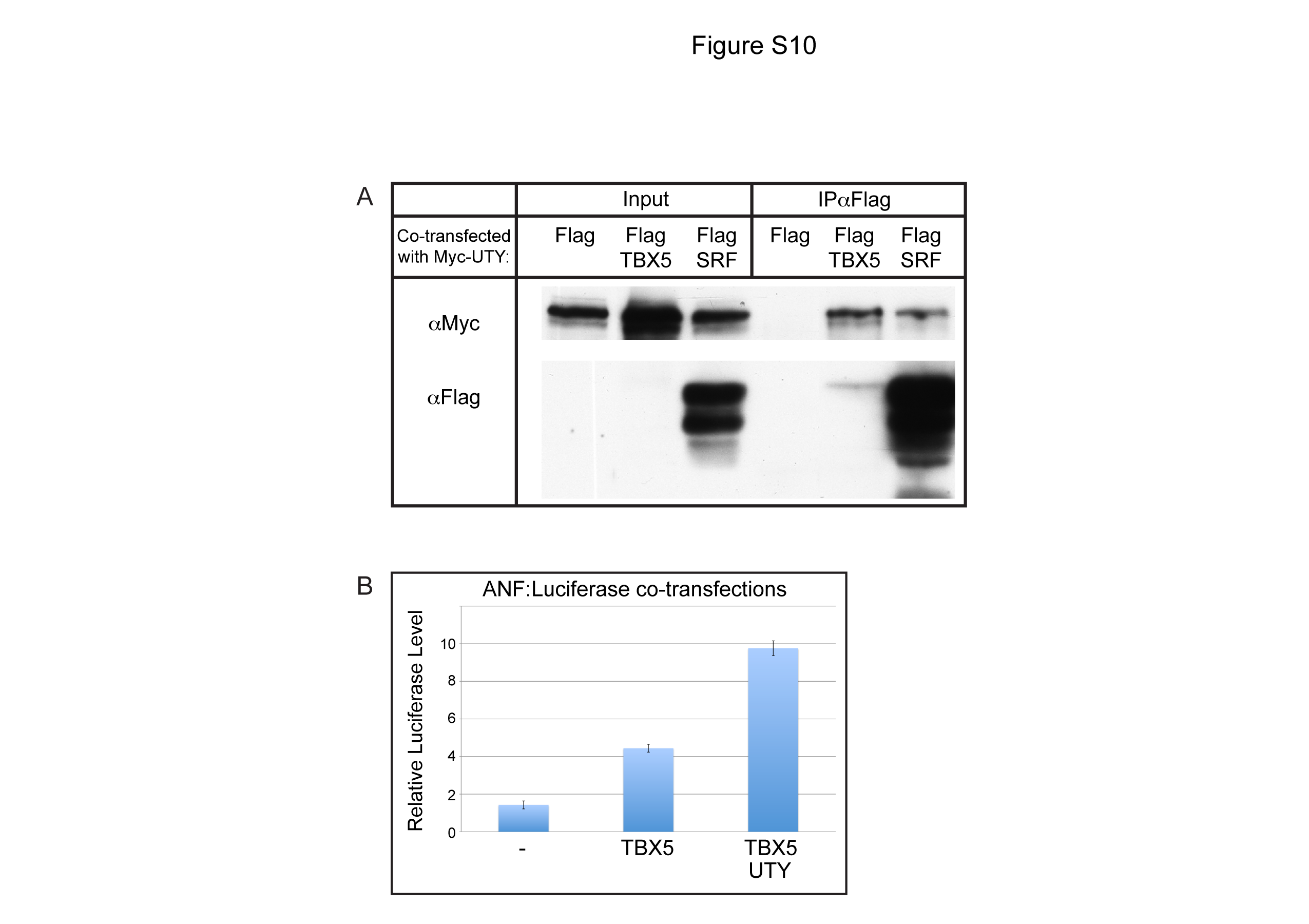

Supplement: Figure S10 — UTX and UTY associate with heart transcription factors and regulate expression of ANF. (A) Myc-UTY was co-transfected with the Flag negative control, Flag-TBX5, or Flag-SRF. Myc-UTY was co-immunoprecipitated by Flag-TBX5 and Flag-SRF. (B) ANF:Luciferase reporter assay. HEK293T were transfected with the reporter ANF:Luciferase construct alone (-), with TBX5, or with TBX5 and UTY. Reporter activity was significantly enhanced with the addition of UTY (t-test p-value = 0.004). (TIF) [file pgen.1002964.s010.tif]
