# Supplementary material for: UTX and UTY Demonstrate Histone Demethylase-Independent Function in Mouse Embryonic Development
Source: PLoS Genet. 2012 Sep 27;8(9):e1002964. doi: 10.1371/journal.pgen.1002964 (PMC3459986; doi:10.1371/journal.pgen.1002964)
Supplement: Table S1 — Utx hemizygous genotype frequency on inbred backgrounds. Observed (Obs) and expected (Ex) frequencies of indicated genotypes (Geno) at embryonic (E) or postnatal (P) developmental stages with χ2 p-values (p-value) for the corresponding crosses to obtain each genotype. At E18.5 on the C57BL/6J background, 5 of the 8 observed XUtxGT1 YUty+ males were on the N8 generation. (DOC) [file pgen.1002964.s011.doc]

Table S1: *Utx* hemizygous genotype frequency on inbred backgrounds.

| **Genotype frequencies of *Utx* hemizygous mutant males** | | | | |
| --- | --- | --- | --- | --- |
| Strain: | 129/SvJ>N2 | | C57BL/6J>N5 | |
| Geno: | X*^UtxGT1^* Y*^Uty+^* | | X*^UtxGT1^* Y*^Uty+^* | |
| Stage: | Obs(Ex) | p-value | Obs(Ex) | p-value |
| E18.5 | 9(13) | 0.12 | 8(13) | 0.02 |
| P25 | 0(14) | <0.01 | 0(15) | <0.01 |
