# Supplementary material for: UTX and UTY Demonstrate Histone Demethylase-Independent Function in Mouse Embryonic Development
Source: PLoS Genet. 2012 Sep 27;8(9):e1002964. doi: 10.1371/journal.pgen.1002964 (PMC3459986; doi:10.1371/journal.pgen.1002964)
Supplement: Table S2 — Genotype frequencies of Sox2Cre driven Utx mutation. Observed (Obs) and expected (Ex) frequencies of indicated genotypes (Geno) at embryonic (E) or postnatal (P) developmental stages with χ2 p-values (p-value) for the corresponding crosses to obtain each genotype. (DOC) [file pgen.1002964.s012.doc]

Table S2: Genotype frequencies of Sox2Cre driven Utx mutation.

| **Genotype frequencies of *Sox2Cre* driven *Utx* mutations** | | | | |
| --- | --- | --- | --- | --- |
| Geno: | X*^Utxfl^* X*^UtxΔ^*, *Sox2Cre* | | X*^Utxfl^* Y*^Uty+^*, *Sox2Cre* | |
| Stage: | Obs(Ex) | p-value | Obs(Ex) | p-value |
| E10.5 | 8( 7) | 0.41 | 7( 7) | 0.80 |
| E18.5 | 0( 8) | 0.00 | 9( 8) | 0.39 |
